# Site-Selective Approaches to Attain Fluorescent Human Insulin Conjugates: Balancing the Site of Labeling and the *In Vivo* Activity

**DOI:** 10.1021/acsomega.4c09498

**Published:** 2025-02-17

**Authors:** Bayan Alkhawaja, Ghayda’ AlDabet, Nour Alkhawaja, Bayan Y. Ghanim, Khaled Al-Khatib, Shaun Reeksting, Andreas Michael, Duaa Abuarqoub, Marwa Mohammad, Andrew G. Watts, Nidal A. Qinna

**Affiliations:** † Faculty of Pharmacy and Medical Sciences, University of Petra, Amman 11196, Jordan; ‡ Department of Life Sciences, University of Bath, Claverton Down, BA2 7AY Bath, U.K.; § University of Petra Pharmaceutical Center, Faculty of Pharmacy and Medical Sciences, Petra University, Amman 11196, Jordan; ∥ Agilent Technologies U.K. Ltd., Lakeside, Cheadle Royal Business Park, Stockport, SK8 3GR Cheshire, U.K.; ⊥ Cell Therapy Center, University of Jordan, Amman 11942, Jordan

## Abstract

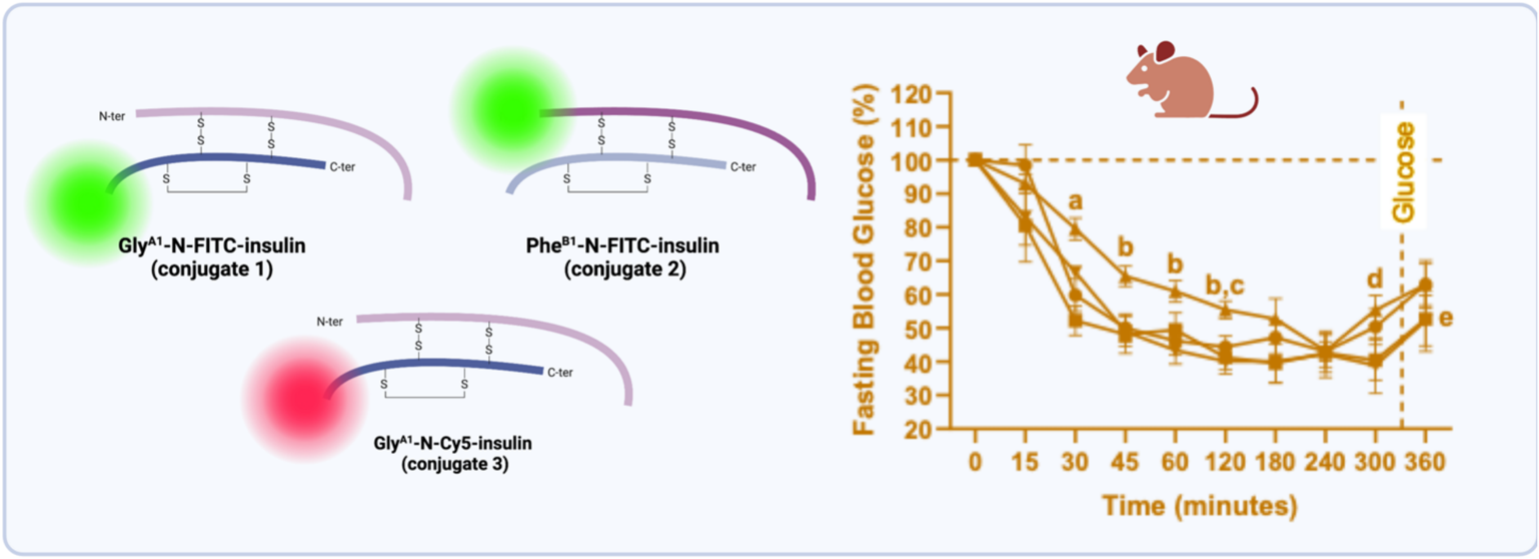

Fluorescent insulin is commonly used for a range of detection and imaging purposes. Achieving site-selective insulin labeling affords superior labeling yield while retaining its biological activity. Insulin labeling is usually achieved using commercial kits with minimal emphasis on the site and degree of labeling. To bridge this gap, this work highlights the essential parameters concerning the development of fluorescent insulin and reflects them on the biological activity of insulin *in vivo*. To this end, monolabeled insulin at the N-terminal of A chain (Gly^A1^-N-FITC-insulin) was prepared using the minimal equivalents of fluorescein isothiocyanate (FITC) dye. In our hands, temperature and pH control were the main parameters affecting the reaction yield, with no dilabeled insulin being attained. To label the N-terminal of the B chain (Phe^B1^-N-FITC-insulin), di*-tert*-butyl decarbonate, known as Boc anhydride, was used before FITC labeling. The attained insulin conjugates, namely, Gly^A1^-N-FITC-insulin and Phe^B1^-N-FITC-insulin, were characterized using protein mass spectroscopy and peptide analysis. A third fluorescent conjugate was prepared using α-haloacetyl-based chemistry. This chemistry’s advantage is maintaining the chain A N-terminal amine basicity, which was essential for its activity. Using α-haloacetyl-based chemistry, azide group-functionalized insulin was prepared, which was further clicked with fluorescent dye affording Gly^A1^-N-Cy5-insulin. According to the *in vivo* efficacy study of the three insulin conjugates, both fluorescent Gly^A1^-N-FITC-insulin and Gly^A1^-N-Cy5-insulin retained the insulin biological activity, suggesting no structural alteration upon the conjugation conditions. Hence, both Gly^A1^-N-FITC-insulin and Gly^A1^-N-Cy5-insulin are effective in labeling and, more importantly, maintaining the *in vivo* activity of insulin. Lastly, *in vitro* binding of Gly^A1^-N-FITC-insulin was successful when it was assayed in NIH/3T3 fibroblast cells. This work has provided facile conjugation approaches for site-specific insulin labeling with dyes or clickable chemistry in conjunction with insulin’s *in vivo* biological activity.

## Introduction

Bioconjugation chemistry aims to modify biological molecules, notably proteins, to attain biological conjugates with added covalent functionalities.^1^ Through the chemical attachment of ubiquitous probes, including biotin,^2^ imaging probes,^3^ and payloads,^4^ bioconjugation chemistry has facilitated the investigation of several biological interactions, the development of biochemical and diagnostic assays, and the development of advanced biotherapeutics.^5,6^ Among bioconjugation applications, fluorescent labeling of biomolecules is a widely applied diagnostic tool. Fluorescence technologies using various fluorescent probes have been used for ample quantitative and qualitative analyses.^7,8^

Failure to control the modification site can lead to losing the biomolecule’s biological function.^9,10^ Hence, site-specific labeling of macromolecules is currently the ultimate goal of conjugation techniques. Despite the challenges related to the specificity of the labeling chemistry and the structure of the target protein, as well as the plausible binding sites,^11^ the advantages of site-specific bioconjugation methods outweigh the random bioconjugation techniques.^12^ Modification of insulin has been comprehensively studied and evaluated to attain site-selective acylation at the nucleophilic amines, depending on either the aqueous buffer pH, the presence of organic solvents, or utilizing protection groups.^13,14^ In addition to the developed enzymatic methods for insulin modification.^15^

Owing to their low toxicity, small size, high detection sensitivity, and other favorable advantages, fluorescence technologies have been widely considered a surrogate for other detection techniques, particularly radioisotopic assays.^16−18^ Fluorescein 5-isothiocyanate (FITC, **1**) is a small chemical fluorophore with low molecular weight (MW 389.4 Da), high molar absorptivity, and detection sensitivity. FITC is a widely adopted probe to fluorescently label macromolecules, particularly proteins, via solvent-accessible amine moieties. FITC forms a covalent bond with amino moieties of biomolecules through its isothiocyanate functional group.^7,19^ FITC is used for various biological applications, such as protein tracing, quantitative and qualitative analyses, protein interaction studying, *in vitro* and *in vivo*, and bioimaging purposes.^20−23^

**Figure 1:**
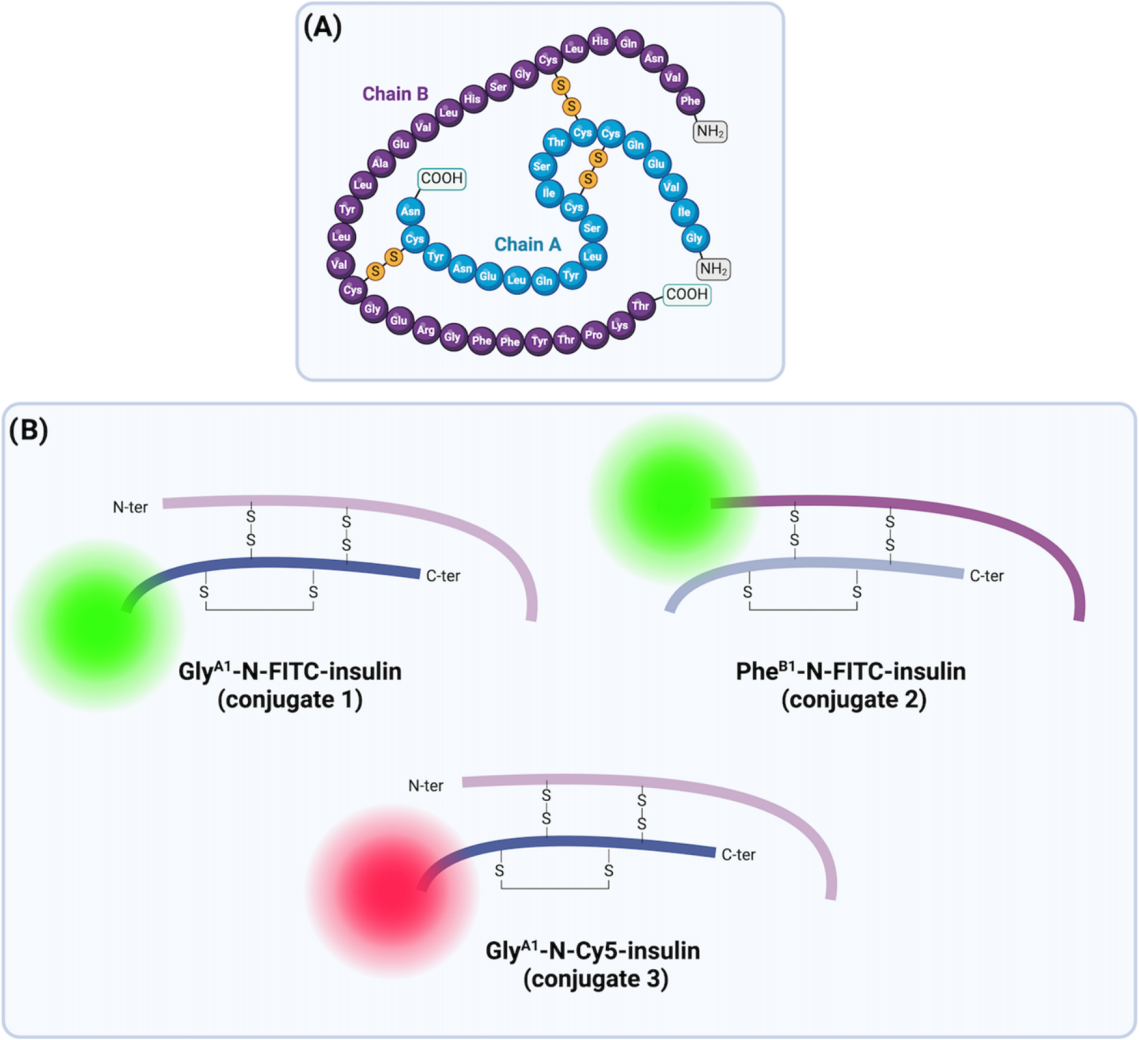
Insulin structure and modification sites. (A) Native human insulin consists of chains A and B, highlighting the three reactive amine moieties. (B) The fluorescent insulin conjugates described in this work. Created in BioRender.com.

FITC-insulin conjugates have been widely described in the literature for versatile biological applications, encompassing biomedical purposes,^24^ binding affinity studies,^25^ drug delivery studies,^26,27^ and analytical applications.^28^ Structurally, human insulin (51 amino acids) consists of 2 chains, A and B chains, connected through 2 interdisulfides and 1 intradisulfide within chain A.^29^ Both N-termini (Gly^A1^-N and Phe^B1^-N) and lysine amino acid (Lys^B29^) represent reacting amine moieties capable of reacting with FITC (Figure 1A). It has been shown that the conjugation at different sites or multiconjugations could affect or mitigate the biological activity of insulin.^28^

Hence, several reports have focused on optimizing the site-specific FITC-insulin conjugation. Hentz et al. described how the conjugation parameters, mainly the pH of the reaction and molar ratios of FITC to insulin, alter the conjugation site and degree of labeling.^30^ Jacob and co-workers considered reaction time a primary parameter determining the degree of labeling.^24^ More recently, Shah and co-workers reported a 4 h conjugation reaction to attain mixtures of insulin adducts by limiting the molar ratio of FITC.^31^

Ample evidence supports the necessity of developing a facile and site-specific protocol to label insulin while maintaining its biological activity. We set out to construct a monolabeled insulin adduct in an adaptable procedure without applying separation techniques. Ultimately, we studied the impact of the conjugation site on the biological activity of insulin *in vivo* coupled with cell labeling *in vitro*. This study showed for the first time the biological activity of fluorescent insulin in a diabetic mouse model (*in vivo*) governed by the labeling site and functionality parameters.

## Results and Discussion

FITC labeling of insulin is mainly achieved through the three plausible and solvent-accessible amine moieties, encompassing N-terminal amines (Gly^A1^-N, Phe^B1^-N-termini) and ε-amine of lysine amino acid (Lys^B29^-N) (Figure 1A). Previously, the construction of dilabeled and trilabeled insulin species was found to significantly mitigate biological activity or completely abolish the activity, respectively.^32^ Moreover, labeling of the ε-amine of lysine amino acid (Lys^B29^-N) was detrimental to insulin activity as only 30% of its activity was maintained.^33,34^ Therefore, developing monolabeled insulin by labeling either Gly^A1^-N or Phe^B1^-N-termini is cardinal for its biological activity.

Herein, we constructed monolabeled insulin adducts using FITC dye (insulin conjugates **1** and **2**). In addition, insulin conjugation with α-haloacetyl-based chemistry followed by click chemistry affording insulin conjugate **3** will be optimized (Figure 1B).

### N-Terminal Labeling to Attain FITC-Insulin

Several reports have highlighted the importance of developing homogeneous FITC-insulin monolabeled adducts. Since insulin possesses three reactive amine moieties (Figure 1A), developing a monolabeled FITC-insulin adduct entailed chromatographic separation methods. Monolabeling with FITC was improved by varying the molar equivalents and incubation time. Nevertheless, unreacted insulin with dilabeled insulin adducts was unavoidable.^24,31^ On the other hand, commercial FITC-insulin is expensive and usually constitutes a mixture of labeled and unlabeled insulin adducts.^35^

In an attempt to construct monolabeled FITC-insulin (conjugate **1**) in a good yield, three main reaction parameters were studied: reaction pH, temperature, and FITC equivalents. The degree of labeling and the main products were evaluated using protein mass spectroscopy (MS), as described in the Supporting Information (Figures S1 and S2).

Insulin possesses three nucleophilic amines for modifications, and due to their difference in p*K*_a_ values, their nucleophilicity in aqueous buffers will be different. For example, most of the nucleophilic e-amine of lysine reacts more rapidly when deprotonated at a high pH (>10.5). The N-terminal amine of chain A (Gly^A1^-N) is expected to be the reactive amine under pH values between 7 and 8. In contrast, most of the nucleophilic e-amine of a lysine amino acid (Lys^B29^-N) is protonated under these conditions. The N-terminal amine of the B chain bears the lowest p*K*_a_ value of 6.8; hence, it is expected to be less reactive than Gly^A1^-N.^14,30^

Hence, we have chosen a neutral pH to perform the conjugation reaction, where the N-terminal amine of chain A is expected to be the target amine and the most reactive nucleophilic amine.

**Figure 2:**
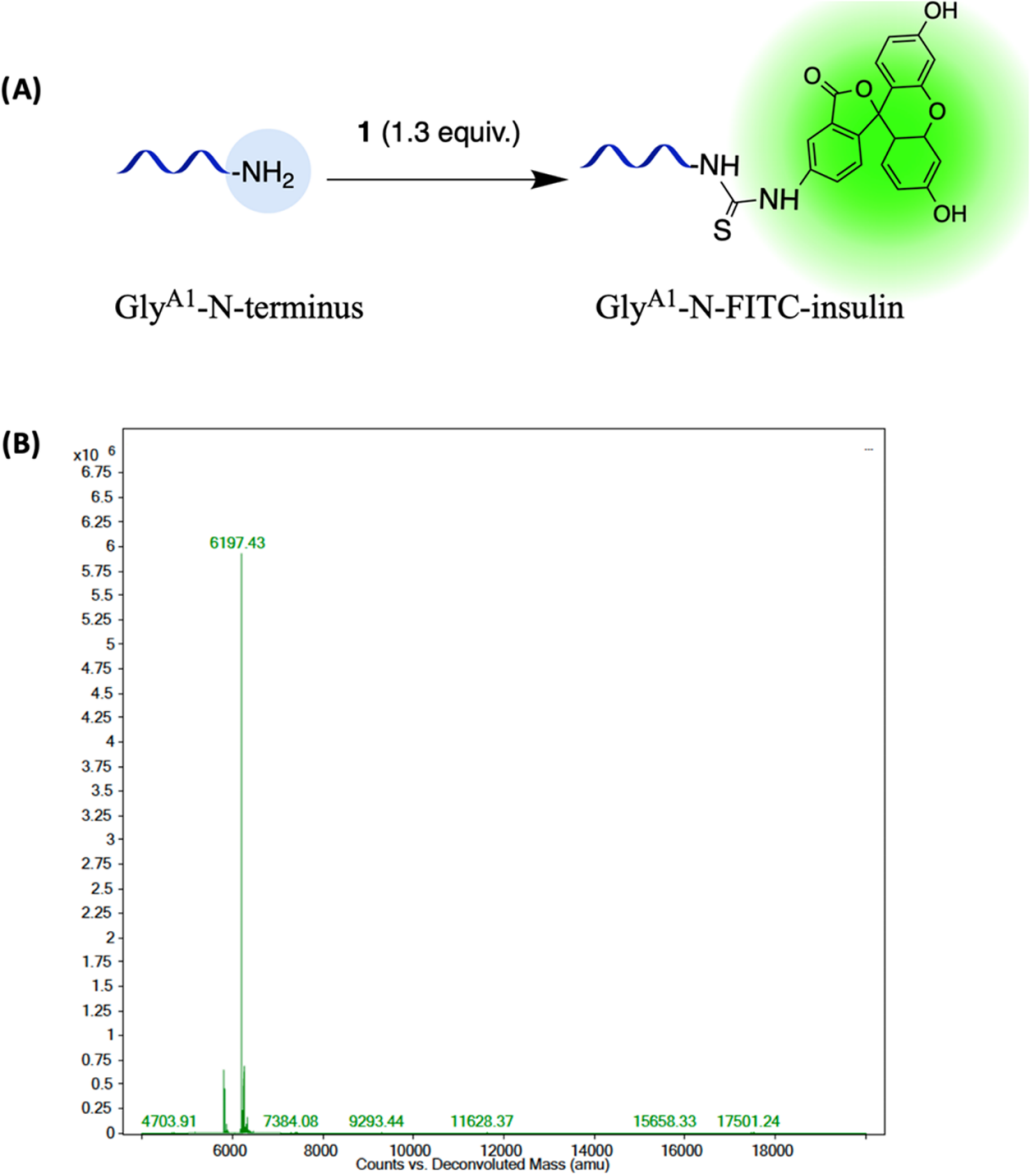
Liquid chromatography-MS (LC-MS) chromatogram of FITC-insulin. (A) The preparation of Gly^A1^-N-FITC-insulin was optimized using limited equivalents of FITC, and (B) the protein MS of Gly^A1^-N-FITC-insulin showed the correct product, 6197 Da.

According to the parameters mentioned above, using a limited equivalent of FITC and controlling the reaction pH and reaction temperature (4 °C) effectively eliminated the higher degree of labeling (di- and tri-FITC adducts). Nevertheless, the yield of the monolabeled product was reduced, as confirmed by MS results (Figure S2). Next, the conjugation reaction was attempted at room temperature (RT) to enhance the yield of the monolabeled product. The main conjugate (Gly^A1^-N-FITC-insulin or insulin conjugate **1**) was successfully confirmed using protein MS and peptide analysis (Figure 2, Table S1, Figure S3). For peptide analysis, insulin was reduced with a reducing agent to separate chains A and B, and the attained mass confirmed chain A’s labeling, and the attained results are in match with the previous findings of Hentz and co-workers.^30^

**Figure 3:**
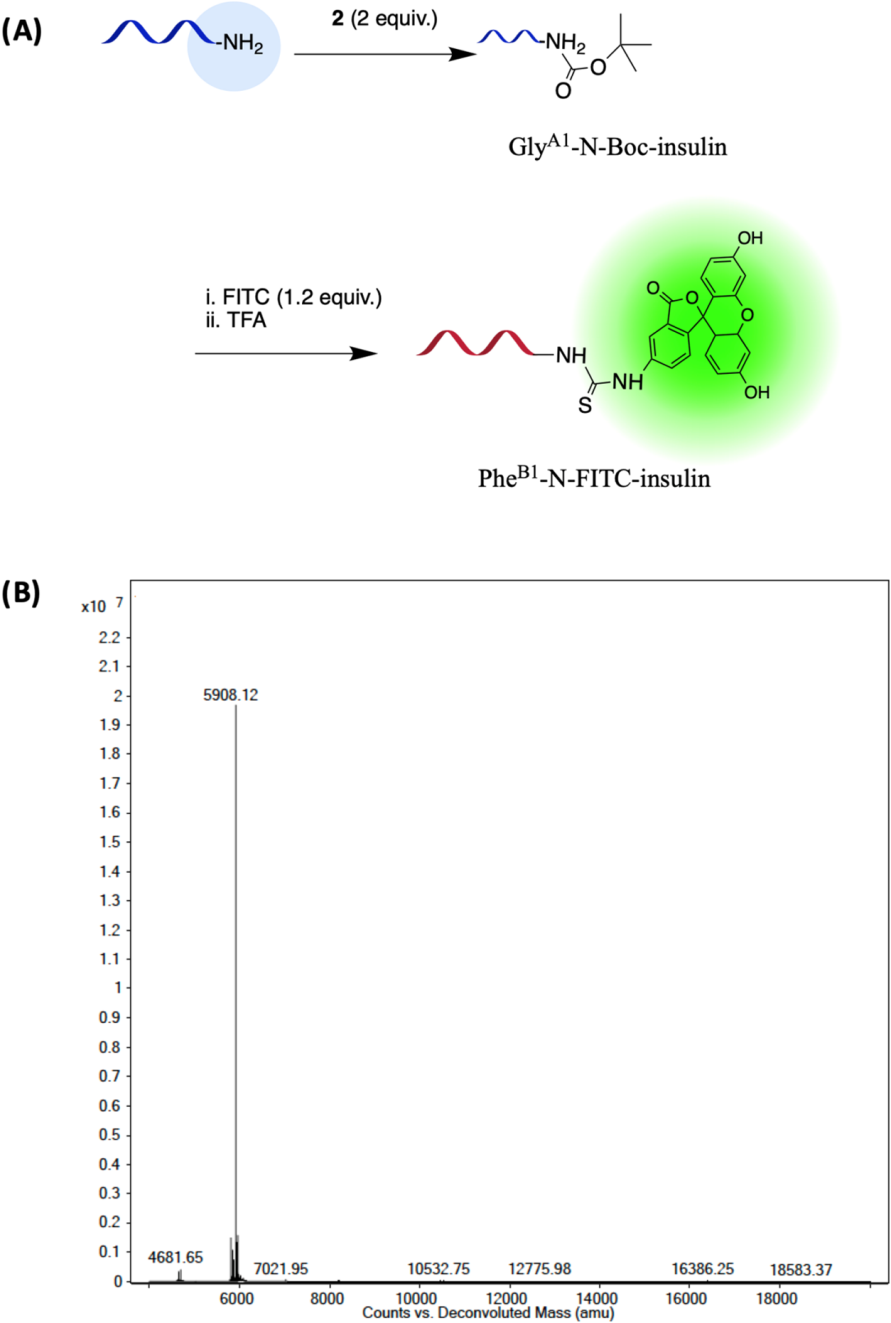
(A) Preparation of Phe^B1^-N-FITC-insulin using Boc anhydride (**2**) and de-Boc steps. (B) Protein MS of Gly^A1^-N-Boc insulin showing the correct product, 5907 Da.

In order to attain monolabeled insulin with the Phe^B1^-N-terminal being the conjugation site, initial protecting of the N-terminal of chain A was attempted with di-*tert*-butyl decarbonate, known as Boc anhydride (**2**) as described previously.^36^ To this end, 1, 2, and 3 mol equiv of Boc anhydride were utilized. According to the MS analysis (Figure S4), implementing 2 mol equiv of Boc anhydride gave better yield and a minor di-Boc Insulin product. Subsequently, the FITC-insulin product was obtained and the product was confirmed by both protein MS and peptide analysis (Figure 3, Table S1). For peptide analysis, insulin was reduced with a reducing agent to separate chains A and B, and the attained mass confirmed chain B’s labeling.

### N-Terminal Labeling to Attain Azide-Insulin and Cy5-Insulin

**Scheme 1:**
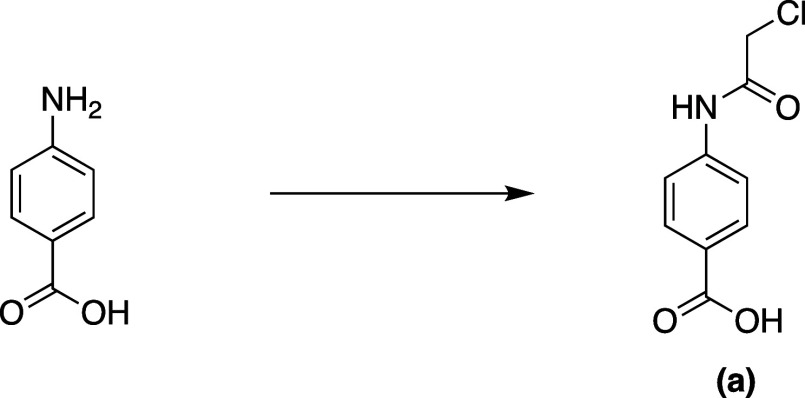
Synthesis of 4-(2-Chloroacetamido)benzoic Acid **a**

**Scheme 2:**
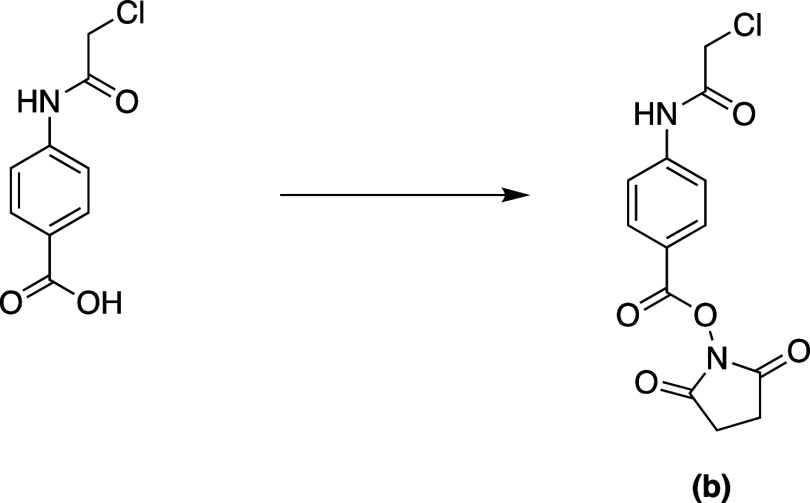
Synthesis of 2,5-Dioxopyrrolidin-1-yl 4-(2-Chloroacetamido)benzoate

**Scheme 3:**
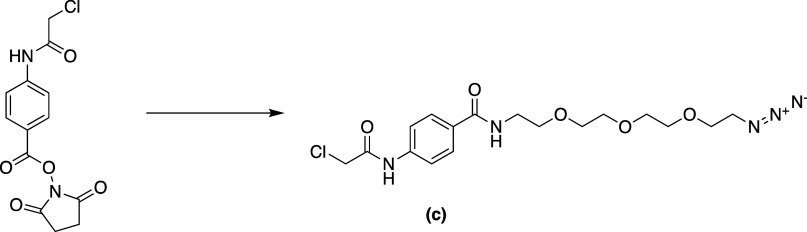
Synthesis of *N*-(2-(2-(2-(2-Azidoethoxy)ethoxy)ethoxy)ethyl)-4-(2-chloroacetamido)benzamide **c**

**Scheme 4:**
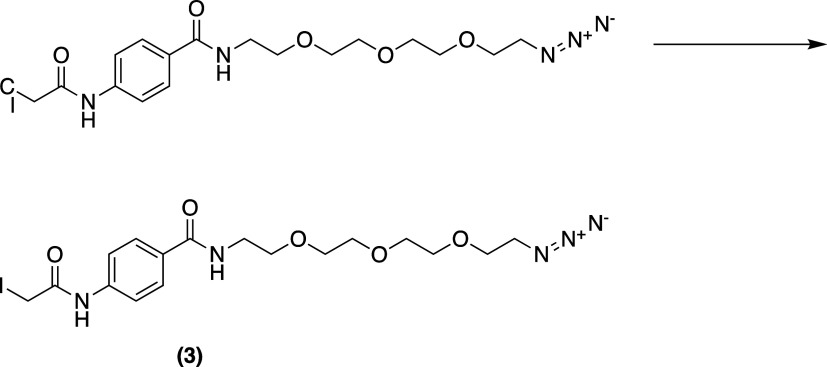
Synthesis of *N*-(2-(2-(2-(2-azidoethoxy)ethoxy)ethoxy)ethyl)-4-(2-iodoacetamido)benzamide **3**

With the developed site-selective monolabeling of FITC-insulin in hand, we set out to construct insulin monolabel with a biorthogonal functional group using α-haloacetyl-based chemistry. The introduction of the azide group (biorthogonal functional group) permits the introduction and preparation of a wide variety of insulin conjugates through click reaction.^37^ To this end, monolabeling of insulin with azide group was performed using 1.3 equiv of *N*-(2-(2-(2-(2-azidoethoxy)ethoxy)ethoxy)ethyl)-4-(2-iodoacetamide)benzamide (**3**). Our method enabled a single-step introduction of biorthogonal groups (azide group) with high specificity and efficiency in native peptides and proteins. This is especially useful for some proteins where genetic incorporation of unnatural amino acids with biorthogonal side chains is difficult. To this end, the synthesis of compound **3** was achieved over four steps, as shown in Schemes 1–4 and Figures S6–S8.

**Figure 4:**
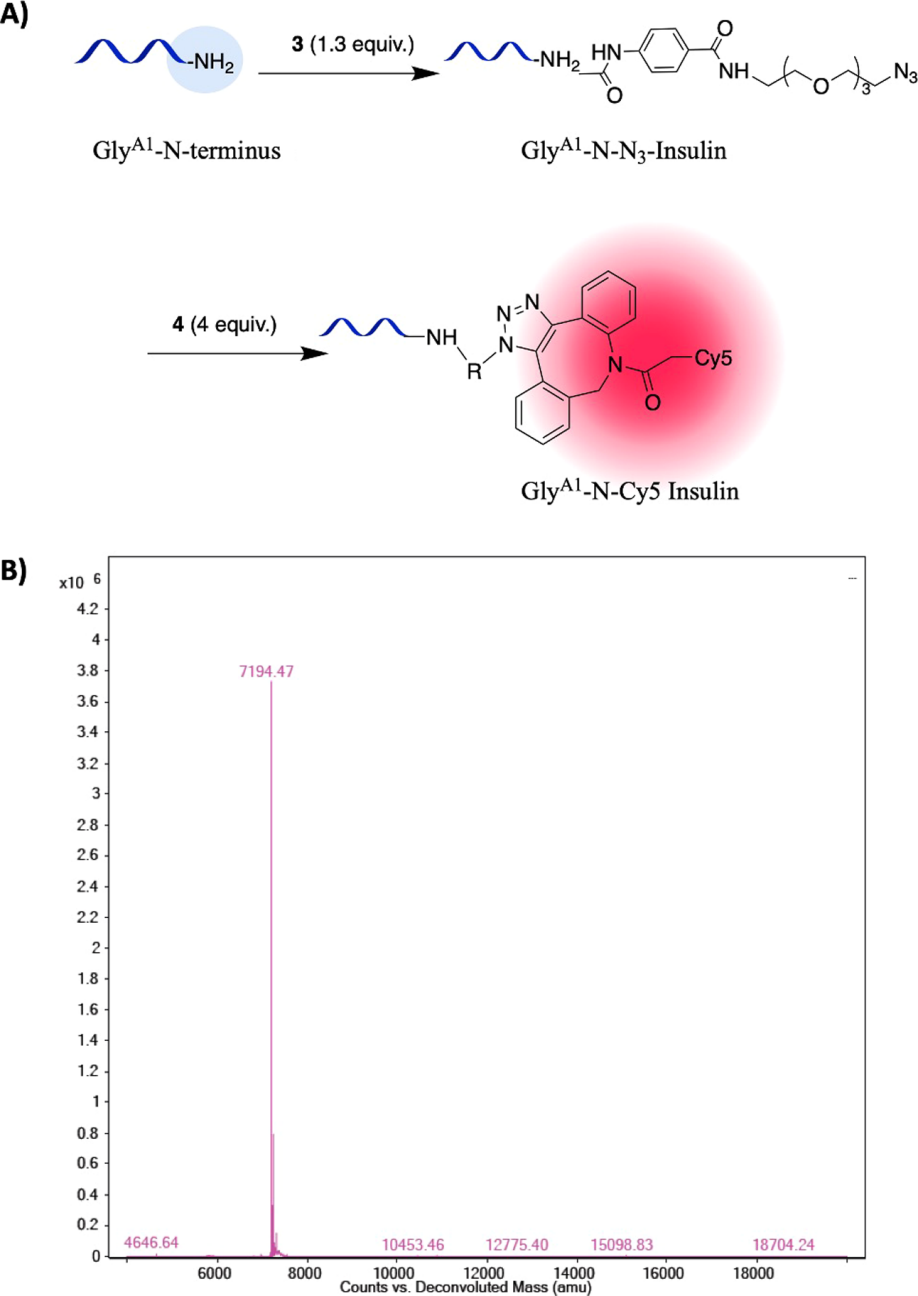
(A) Preparation of insulin conjugate **3** (Gly^A1^-N-Cy5). (B) Protein MS of Gly^A1^-N-Cy5-insulin showing the correct product at 7194 Da.

It was demonstrated that the acylation of Gly^A1^-N exhibited a 5-fold decrease in activity compared with its reductive alkylation of Gly^A1^-N.^38^ Hence, our described one-step procedure permits the construction of labeled insulin while retaining the Gly^A1^-N-terminal basicity and overall insulin activity (Figure 4).

Subsequently, the click reaction between Gly^A1^-N-N_3_-insulin and DBCO-Cy5 reagent (**4**) gave Gly^A1^-N-Cy5-insulin in almost 100% yield as no azide adduct was detected by protein MS (Figure 4).

Near-infrared (NIR) fluorescence imaging, with a 650–900 nm wavelength range, is widely used for noninvasive *in vivo* biomedical imaging of specific targets. Being of low background autofluorescence and high tissue transparency, NIR fluorescence probes have been commonly used for ubiquitous diagnostic purposes.^39−41^ The widely used Cy5 dye^42,43^ was conjugated to insulin, which could be adopted for noninvasive *in vivo* imaging studies.

**Figure 5:**
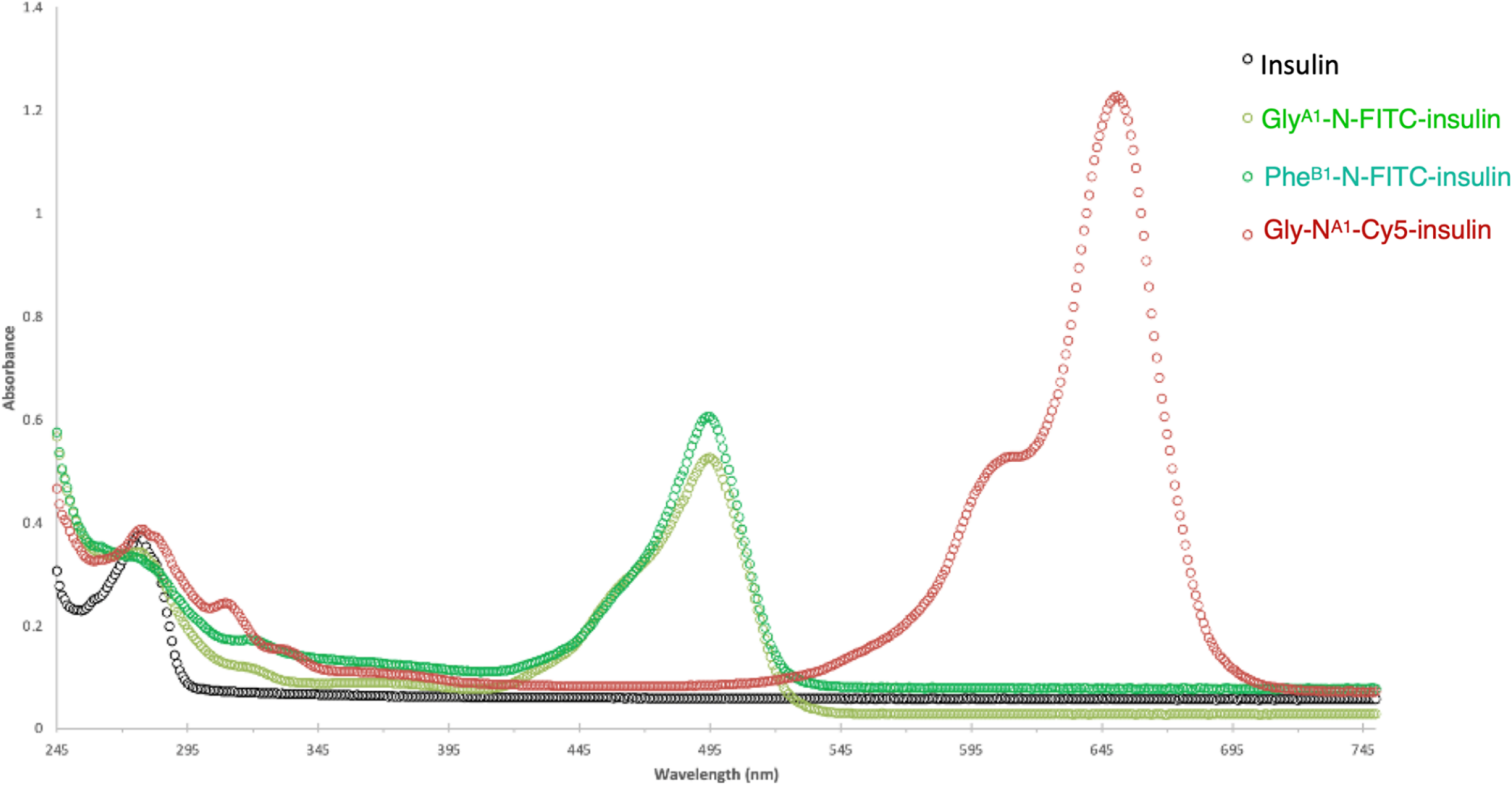
UV–visible absorbance spectra of insulin and insulin fluorescent conjugates (**1**–**3**).

It is worthwhile to mention that the prepared conjugates were subjected to a simple purification procedure to remove unreacted reagents. Next, for comparison purposes, UV–visible spectra of the three conjugates were obtained along with insulin. Following this, conjugates **1** and **2** displayed an λ_max_ at 495 nm, corresponding to FITC dye. Conjugate **3** showed λ_max_ at 646 nm, corresponding to Cy5 dye (Figure 5). Next, the aggregation index (AI) was calculated for insulin fluorescent conjugates and compared with that of insulin. The calculated AI% for the conjugates were 37, 79, and 53%, respectively. On the other hand, the insulin sample showed an AI value of 24%. Generally, AI above 10% represents the presence of significant aggregation.^44,45^ To this end, sufficient soluble aggregates were found across the conjugates, which refer to the increasing potential of insulin aggregation upon exposure to conjugation conditions, particularly Phe-N^B1^-FITC-insulin.

Lastly, Table S1 and Figure S5 summarize all of the prepared conjugates in this work, along with their expected and found MS results.

### *In Vivo* Efficacy of Labeled Insulin in Diabetic Mice

The efficacy of the prepared insulin bioconjugates was tested on diabetic animals, developed using STZ, and the circulating glucose concentration was monitored throughout a 5 h time frame, followed by challenging the blood glucose levels with a moderate glucose dose.^46^

**Figure 6:**
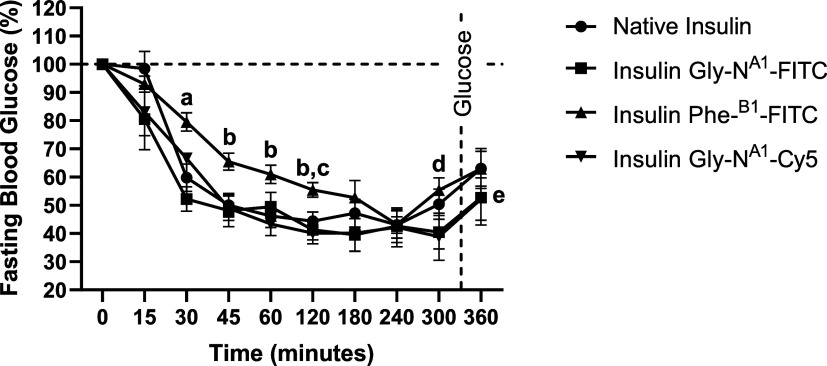
Percentage change in fasting blood glucose levels in diabetic animals. Percent changes in fasting blood glucose of animals treated with 0.5 IU/kg control recombinant human insulin, Gly^A1^-N-FITC-insulin, Phe-N^B1^-FITC-insulin, and Gly^A1^-N-Cy5-insulin were plotted by the elapsed time after administration. Data points are expressed as the mean ± standard error of the mean (SEM) (*n* = 7). (a) Significant difference between Phe-N^B1^-FITC-insulin and all other groups (*p* ≤ 0.05 in comparison to native and Gly ^A1^-N-Cy5-insulin; *p* ≤ 0.01 in comparison to Gly^A1^-N-FITC-insulin); (b) significant difference between Phe^B1^-N-FITC-insulin and Gly^A1^-N-Cy5-insulin (*p* ≤ 0.05); (c) significant difference between Phe-N^B1^-FITC-insulin and Gly^A1^-N-FITC; (d) significant difference in the glucose level in comparison to 0-time reading in all groups (*p* ≤ 0.01); (e) significant difference in the glucose level in comparison to 0-time reading of Gly^A1^-N-FITC and Gly^A1^-N-Cy5-insulin treated groups (*p* ≤ 0.05).

Comparable blood glucose levels were noticed in both groups that received Gly^A1^-N-FITC and Gly^A1^-N-Cy5 over 5 h, as well as when compared to the native insulin (Figure 6). Nonetheless, groups treated with both Gly^A1^-N insulin bioconjugates exhibited a prompt response in blood glucose reduction within 15 min, unlike the native insulin, which expressed a delayed response detected after 30 min. On the other hand, animals treated with Phe-N^B1^-FITC showed a significantly slower response in blood glucose reduction throughout the first 2 h from treatment compared to other groups, as shown in Figure 6. All groups reached a maximum reduction of blood glucose after 240 min from treatment and a rebounce in blood glucose was observed later on, which is possibly due to the endogenous glycemic homeostasis.^47^ In order to challenge glycemic control and the reflux of blood glucose levels after insulin administration, mice were dosed with a moderate dose of glucose.^48^ The subsequent glucose reading indicated that all of the conjugates displayed similar pharmacological behavior to the native insulin, preventing a rapid spike in blood glucose after bolus glucose intake. The increase in glycemic levels from the glucose dose was rapid; however, clinically and statistically insignificant compared to the glucose reading collected before sugar intake. Taken together, it was confirmed that the conjugates were significantly effective in controlling glucose levels in diabetic models, up to 300 min (*p* ≤ 0.01) and after sudden intake of sugar in animals treated with Gly-N^A1^-FITC and Gly-N^A1^-Cy5-insulin (*p* ≤ 0.05). Extending the blood glucose monitoring duration could potentially provide a more comprehensive understanding of its long-term effect.

### Evaluation of Cellular Binding Using Gly-N^A1^-FITC-Insulin

**Figure 7:**
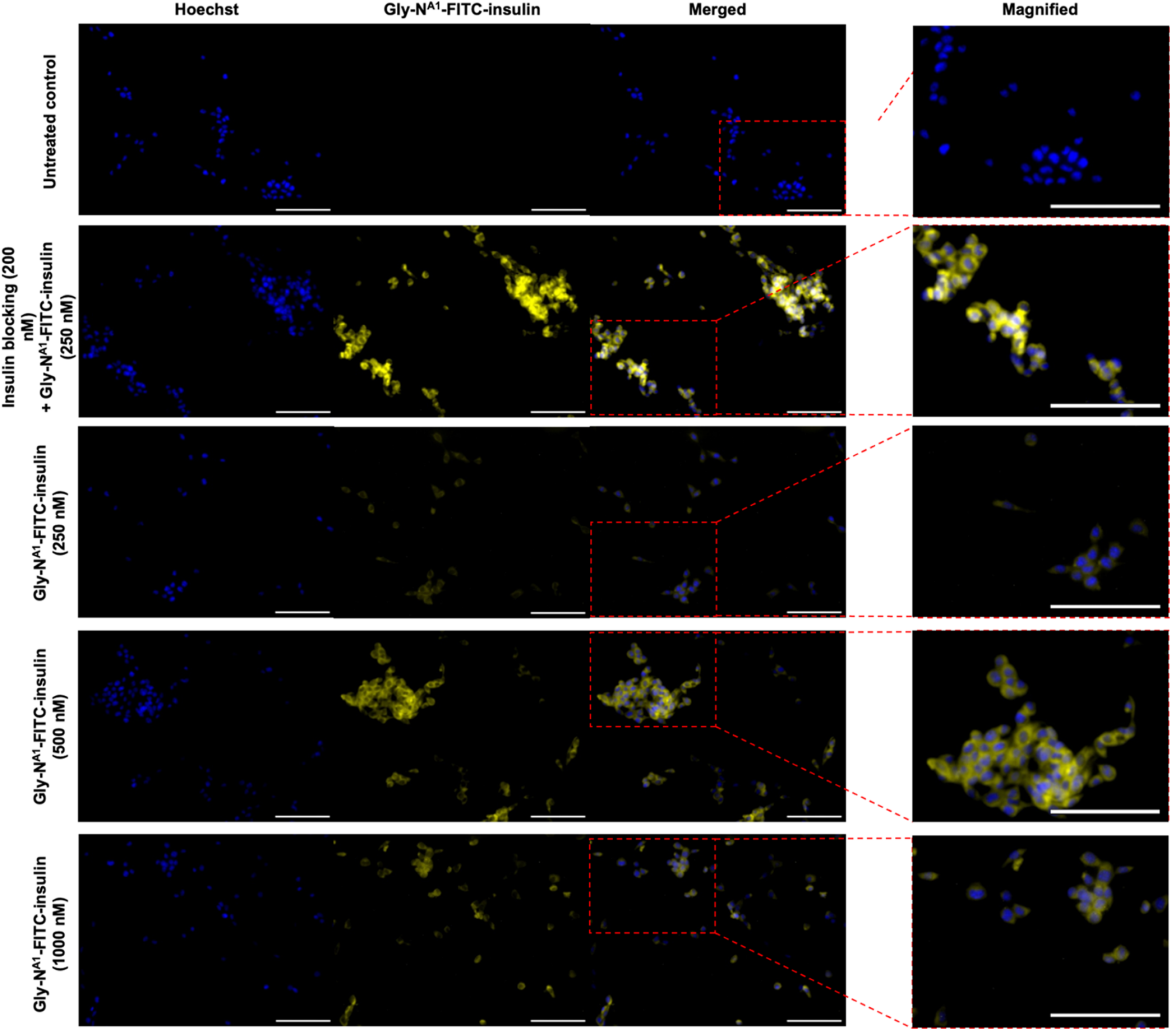
Cell labeling using an increasing concentration of fluorescent insulin conjugate **1** (Gly^A1^-N-FITC-insulin). All images were acquired at 20× using a CELENA X High Content Imaging System at 20×. The scale bar was 100 μm. Nuclear stain Hoechst 33342 (blue). Gly^A1^-N-FITC-insulin (yellow).

Lastly, cellular binding of labeled insulin preparations was demonstrated in NIH/3T3 cells. Cells were incubated with Gly^A1^-N-FITC-insulin at increasing concentrations and an insulin-blocked sample was included by prior incubation of cells with native insulin (200 nM) before treatment with conjugated insulin. As shown in Figure 7, consistent and efficient binding is seen by using 500 and 1000 nM. However, despite blocking the cells with native insulin for 10 min prior to incubation with Gly^A1^-N-FITC-insulin (250 nM) for another 10 min, an abundant fluorescence signal was observed, suggesting potential complexing with native insulin. Nevertheless, the fluorescent conjugate Gly^A1^-N-FITC-insulin successfully labeled cells in a concentration-dependent manner, thus indicating efficient binding to insulin receptors.

In conclusion, this study presents a simple and adaptable method for achieving monolabeled fluorescent insulin using minimal amounts of fluorescent dye without the need for separation techniques. The N-terminal monolabeling of insulin with FITC dye was accomplished by utilizing lower dye equivalents. This research has established a connection between the binding sites and the *in vivo* activity of insulin. Additionally, insulin-bearing azide was prepared, followed by a click reaction to produce fluorescent insulin. The insulin conjugates Gly^A1^-N-FITC and Gly^A1^-N-Cy5 retained their *in vivo* activity after the conjugation conditions, indicating that no significant structural changes occurred. Therefore, both conjugates, prepared with FITC dye and using α-haloacetyl-based chemistry for Cy5 conjugation, are effective for labeling and, importantly, for maintaining the *in vivo* activity of insulin.

## Material and Methods

### Material

Recombinant human (rh) insulin powder was purchased from Biocon (Bangalore, India). Labeling chemicals, including fluorescein isothiocyanate isomer I (FITC), di-*tert*-butyl dicarbonate (Boc anhydride), and dibenzocyclooctyne-Cy5 (DBCO-Cy5) were obtained from Sigma-Aldrich. Anhydrous solvents were purchased from Acros Organic. *N*-(3-(Dimethylamino)propyl)-*N*′-ethylcarbodiimide hydrochloride, *N*-hydroxysuccinimide, and 2-(2-azidoethoxy)ethan-1-amine were purchased from Tokyo Chemical Industry Co., Ltd. (TCI), Japan. Deuterated solvents for NMR were purchased from Cambridge Isotope Laboratories.

Dulbecco’s modified Eagle’s medium (EuroClone, Netherlands), supplemented with penicillin/streptomycin (Biowest), l-glutamine (Biowest), nonessential amino acid (EuroClone, Netherlands), and fetal bovine serum (FBS) (Cytiva). TripLE Enzymes and Nuclear stain Hoechst 33342 were purchased from Thermo Fisher Scientific (Middletown, VA). The chemical structures and properties of the adopted reagents are illustrated in Table 1.

**Table 1:**
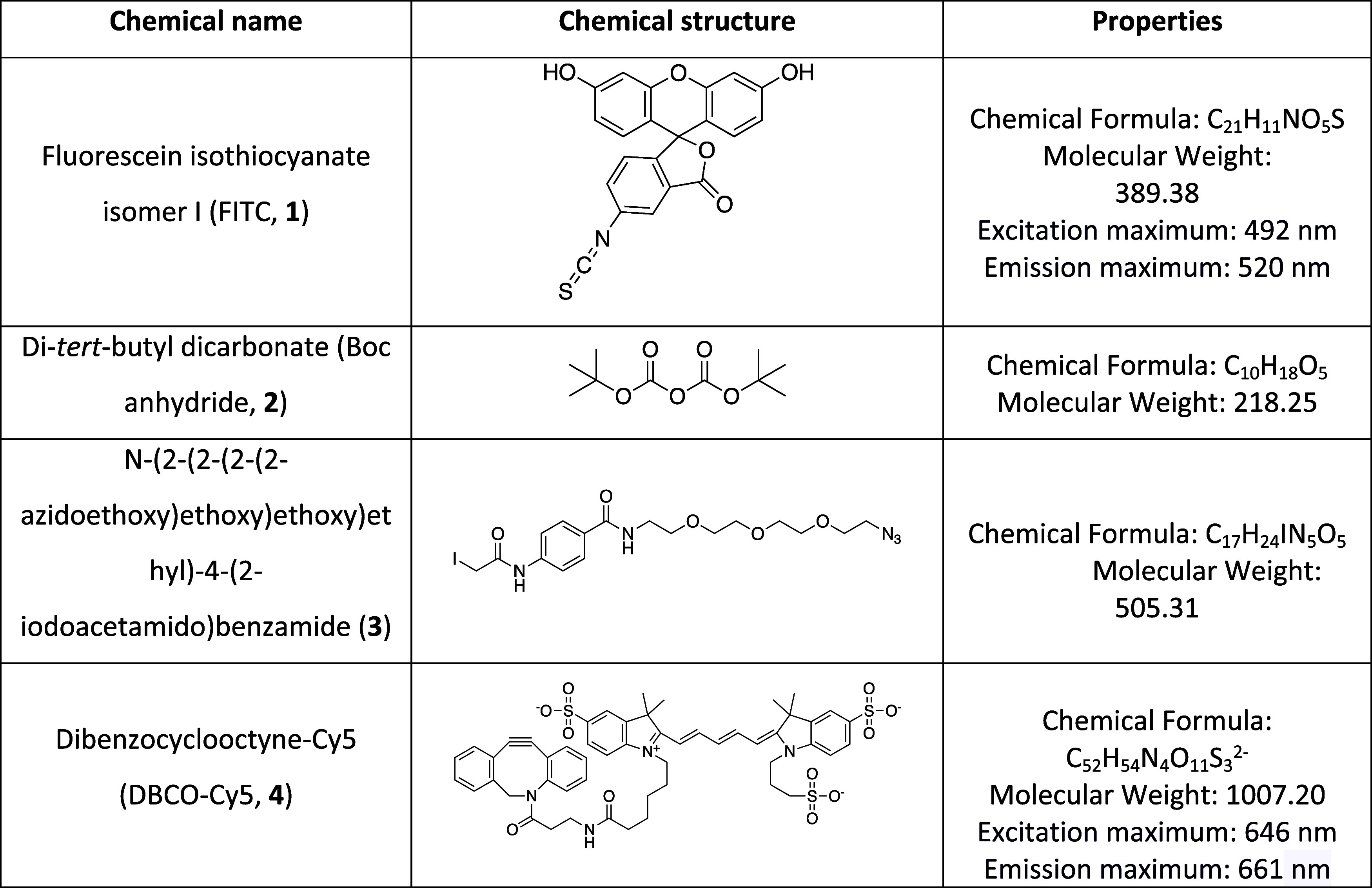
Chemical Structures and Properties of the Adopted Reagents in This Work

### Synthesis of *N*-(2-(2-(2-(2-Azidoethoxy)ethoxy)ethoxy)ethyl)-4-(2-iodoacetamido)benzamide (**3**)

#### 4-(2-Chloroacetamido)benzoic Acid

A cooled solution of 4-aminobenzoic acid (1.0 g, 7.3 mmol) in anhydrous tetrahydrofuran (THF) was prepared, and then 2-chloroacetyl chloride (0.91 g, 8.0 mmol, 1.1 equiv) was added dropwise. The mixture was then stirred at room temperature for 2 h. The obtained precipitate was filtered, washed with water, and then completely dried under a high vacuum to give 4-(2-chloroacetamido)benzoic acid **a** as a white precipitate (1.4 g, 90%). ^1^H NMR (400 MHz, THF-*d*_8_) δ 9.51 (s, 1H), 7.87 (dd, *J* = 100.8, 8.8 Hz, 4H), 4.18 (s, 2H). ^13^C NMR (101 MHz, THF) δ: 164.24, 162.54, 140.75, 128.58, 124.29, 116.56, 41.13. ESI-HRMS: Expected for C_9_H_9_Cl_1_N_1_O_3_ (M + H^+^) = *m*/*z* 214.0265. Found: *m*/*z* 214.0257.

#### 2,5-Dioxopyrrolidin-1-yl 4-(2-Chloroacetamido)benzoate

*N*-(3-Dimethylaminopropyl)-*N*′-ethylcarbodiimide hydrochloride, 1-ethyl-3-(3′-dimethylaminopropyl)carbodiimide·HCl (EDC·HCl, 0.99 g, 5.16 mmol, 1.1 equiv), and *N*-hydroxysuccinimide (0.59 g, 5.16 mmol, 1.1 equiv) were dissolved in dimethylformamide (DMF). Then, DMF solution was added to a stirred solution of the acid (1 g, 4.69 mmol, **a**) in THF. The reaction mixture was then stirred at room temperature for 4 h before being concentrated under reduced pressure. The residue was subjected to workup, and the solvent was evaporated under reduced pressure. The crude was used as such without further purification. The product was confirmed using ESI-HRMS. ESI-HRMS: Expected for C_13_H_11_Cl_1_N_2_O_5_Na (M + Na^+^) = *m*/*z* 333.0249 and found: *m*/*z* 333.0304.

#### *N*-(2-(2-(2-(2-Azidoethoxy)ethoxy)ethoxy)ethyl)-4-(2-chloroacetamido)benzamide

2-(2-Azidoethoxy)ethan-1-amine (0.46 g, 2.10 mmol, 1.3 equiv) was added to a solution of 2,5-dioxopyrrolidin-1-yl 4-(2-chloroacetamido)benzoate **b** (0.50 g, 1.61 mmol) in anhydrous THF. The reaction mixture was then stirred at room temperature for 1 h before being concentrated under reduced pressure. The obtained residue was dissolved in dichloromethane (DCM) and subjected to workup, and the solvent was evaporated under reduced pressure. The crude was further purified by silica gel chromatography (5% methanol/DCM) to give *N*-(2-(2-(2-(2-azidoethoxy)ethoxy)ethoxy)ethyl)-4-(2-chloroacetamido)benzamide **c** as a white solid (0.545 g, 68%). ^1^H NMR (400 MHz, CDCl_3_) δ 8.28 (s, 1H), 7.93–7.69 (m, 2H), 7.66–7.50 (m, 2H), 6.67 (s, 1H), 4.15 (s, 2H), 3.80–3.43 (m, 14H), 3.37–3.19 (m, 2H). ^13^C NMR (101 MHz, CDCl_3_) δ: 166.44, 128.14, 119.36, 70.63, 70.52, 70.22, 69.96, 69.70, 50.61, 42.77, 39.73. HRMS: Expected for C_17_H_25_ClN_5_O_5_ (M + Na^+^) = *m*/*z* 436.1364. Found: *m*/*z* 436.1395.

#### *N*-(2-(2-(2-(2-Azidoethoxy)ethoxy)ethoxy)ethyl)-4-(2-iodoacetamido)benzamide

To a stirring solution of *N*,*N*′-(4-((2-(2-(2-(2-azidoethoxy)ethoxy)ethoxy)ethyl)carbamoyl)-1,3-phenylene)bis(2-chloroacetamide) **c** (0.5 g, 1.21 mmol) in dry acetonitrile (ACN, 20 mL) was added KI (0.80 g, 4.84 mmol, 4 equiv). The mixture was refluxed for 5 h. The obtained mixture was filtrated, and the solvent was evaporated under reduced pressure. The crude was purified by silica gel chromatography: (50% acetone/chloroform) to give *N*-(2-(2-(2-(2-azidoethoxy)ethoxy)ethoxy)ethyl)-4-(2-iodoacetamido)benzamide **3** as a yellow solid (0.47 g, 77%). ^1^H NMR (400 MHz, CDCl_3_) δ 8.10 (s, 1H), 7.72 (d, *J* = 8.7 Hz, 2H), 7.54 (d, *J* = 8.7 Hz, 2H), 6.76 (s, 1H), 3.83 (s, 2H), 3.74–3.42 (m, 14H), 3.32 (dd, *J* = 5.6, 4.5 Hz, 2H). ^13^C NMR (101 MHz, CDCl_3_) δ: 166.80, 165.36, 151.86, 140.25, 128.08, 119.22, 70.52, 70.42, 70.19, 69.88, 50.61. ESI-HRMS: Expected for C_17_H_25_IN_5_O_5_ (M + H^+^) = *m*/*z* 506.0895. Found: *m*/*z* 506.0915.

### Preparation of the N-Terminus FITC-Insulin

Insulin (5 mg) was dissolved in 500 μL of 0.01 N HCl. The pH was adjusted to around 7 using 1 N NaOH solution and 150 μL of 50 mM Tris·HCl buffer (pH 7) was added.

### Gly^A1^-N-FITC-Insulin (Insulin Conjugate **1**)

Fluorescein isothiocyanate isomer I (FITC, **1**) solution was prepared in DMSO.

To 1 equiv of insulin in conjunction buffer (pH 7), 1.3 equiv of FITC (**1**) was added, and the reaction was protected from light and held overnight at room temperature (RT).

### Phe^B1^-N-FITC-Insulin (Insulin Conjugate **2**)

Di-*tert*-butyl decarbonate (Boc anhydride **2**) solution was prepared in DMSO.

To 1 equiv of insulin in conjunction buffer (pH 7), 2 equiv Boc anhydride (**2**) was added and incubated for 6 h at 37 C; subsequently, FITC (**1**) was added, and the reaction was held overnight at RT (protected from light). The sample was treated with trifluoroacetic acid (TFA) for 3 h at RT to remove the attached Boc group.^36^

### Gly^A1^-N-Cy5-Insulin (Insulin Conjugate **3**)

Preparation, purification, and characterization of *N*-(2-(2-(2-(2-azidoethoxy)ethoxy)ethoxy)ethyl)-4-(2-iodoacetamido)benzamide are described above.

To 1 equiv of insulin in conjunction buffer (pH 7), 1.3 equiv of *N*-(2-(2-(2-(2-azidoethoxy)ethoxy)ethoxy)ethyl)-4-(2-iodoacetamido)benzamide (azide-linker **3**) was added and incubated overnight at RT, subsequently, 4 equiv of dibenzocyclooctyne-Cy5 **4** (DBCO-Cy5, **4**) was added, and the reaction was held overnight at RT.

### Desalting of the Conjugation Reactions from Unreacted Reagents

The attained conjugates (products) were then purified using PD-10 desalting columns packed with Sephadex G-25 resin (CyticaÒ). Briefly, the reaction mixture was made up of 2.5 mL and applied to the columns. Fresh Tris·HCl buffer (pH 7) was used to elute the mixture from the column, and the eluent was collected. The conjugate fractions were combined and concentrated using an ultracentrifugal filter (3000 MWCO).

### Protein Mass Spectroscopy MS and Peptide Analysis

An Agilent QTOF 6545 (Jetstream ESI spray source coupled to an Agilent 1260 Infinity II Quat pump HPLC, 1260 autosampler, column oven compartment, and variable wavelength detector) was used for LC-MS analysis. For intact protein analysis, the MS was operated in positive ionization mode with the gas temperature at 350 °C, drying gas flow at 11 L/min, and nebulizer gas flow at 50 psi (3.44 bar). The sheath gas temperature and flow were set to 400 °C and 12 L/min, respectively. Data analyses were performed in MassHunter BioConfirm version 10.0.

For peptide analysis, the MS was operated in positive ionization mode with the gas temperature at 250 °C, the drying gas at 13 L/min, and the nebulizer gas at 45 psi (3.10 bar) in the 50–2500 *m*/*z* range, collecting 1 spectrum/s. The VCap, Fragmentor, and Skimmer were set to 3500, 125, and 45, respectively. The sheath gas temperature and flow were set to 350 °C and 12 L/min, respectively. Chromatographic separation was performed on a Water Acquity BEH C18 2.1 mm × 50 mm, 1.7 μm using H20 (Merck, LC-MS grade) with 0.1% formic acid (FA, Fluka) v/v and acetonitrile (ACN, VWR, HiPerSolv) with 0.1% FA v/v as mobile phases A and B, respectively. The column was operated at a flow rate of 0.3 mL/min at 50 °C starting with 5% mobile phase B for 0.5 min; the gradient was set to 100% B at 4 min, held at 100% B for 1.1 min, and then returned to 5% B at 6.0 min in a total 9 min run time. Five microliter injections of the samples were made. The variable wavelength detector was set to collect wavelengths of 280 and 320 nm at 2.5 Hz. Data processing was automated in Qual B 07.00 with a Find by formula matching tolerance of 10 ppm.

Samples were exchanged into a deionized water ultracentrifugal filter (3000 MWCO) and analyzed to get intact protein MS or incubated with 2 equiv of tris(2-carboxyethyl)phosphine (TCEP) (reducing agents) for peptide analysis.

### UV–Visible Spectroscopy

UV–visible spectroscopy (245–750 nm) was recorded by using a plate reader (Thermo Scientific Multiskan Sky). The aggregation index (AI) value of insulin conjugates was calculated using the following equation
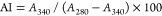


### Experimental Animals

8–10 weeks male Balb/c mice weighing 30 (±5) g were housed at the University of Petra Pharmaceutical Center Laboratory Animal Research Unit under controlled environmental conditions: temperature (24 (±3) °C), relative humidity (55–65%), and artificial photoperiodic light cycle (12 h light/dark). Mice were acclimated for 10 days before the experiment day and were given standard rodent chow (Jordan Feed Co. Ltd., Amman, Jordan) and clean reverse osmosis (RO) water *ad libitum*. All animal procedures were conducted in compliance with the University of Petra Animal Care Guideline, which complies with the Federation of European Laboratory Animal Science Association guidelines (FELASA). The study protocol was reviewed and approved by the Ethical Committee (Ethical approval code: (E/A/1/2023)).

### Induction of Diabetes Model Using STZ

Animals were divided randomly into two main groups: a nondiabetic control group that received citrate buffer and a diabetic group where diabetes was induced by a single i.p. dose of 175 mg/kg of streptozotocin (STZ) (Cayman Cat: CAY13104) freshly prepared in a citrate buffer (pH 4.5). Accordingly, animals were considered diabetic when fed blood glucose levels exceeded ≥300 mg/dL. After ensuring diabetes development, animals were further divided into four groups, each of seven animals: a native insulin-treated group and three other groups treated with candidate insulin bioconjugates, namely, Gly-N^A1^-FITC-insulin, Phe-N^B1^-FITC-insulin, and Gly-N^A1^-Cy5-insulin.

### *In Vivo* Determination of Insulin Bioactivity

Insulin bioactivity was measured against native human insulin based on its ability to induce a hypoglycemic effect following subcutaneous injection in diabetic mice.^49−51^ Mice were fasted for 4 h before the test with wire mesh bottoms and provided with drinking water *ad libitum*. At time 0, the control group was injected with 0.5 IU/kg of native insulin subcutaneously. Whereas the treatment groups were injected with equivalent doses of either conjugate **1** (insulin Gly-NA1-FITC), conjugate **2** (insulin Phe-B1-FITC), or conjugate **3** (insulin Gly-NA1-Cy5). After baseline glucose testing, blood glucose levels were determined via tip-tail sampling at 15, 30, 45, 60, 120, 180, 240, and 300 min post insulin bioconjugate administration and native insulin using Stanom glucometer (NC). Further testing of insulin bioactivity was made by challenging the mice with a single oral glucose dose (100 mg/mouse) 300 min after insulin injection, followed by measuring blood glucose reading at 360 min.^47^

### *In Vitro* Cell Binding and Labeling

Cells were seeded at a 60–70% cell confluence on coverslips placed in 6-well plates and cultured for 24 h. Cells were then incubated with Gly-N^A1^-Cy5-insulin at concentrations of 250, 500, and 1000 nM for 10 min. A Hu-insulin-blocked sample was included by incubating the cells with Hu-insulin (200 nM) (in Tris buffer) for 10 min prior to treatment with conjugated insulin (250 nM). Coverslips were then flipped on a glass slide mount with Fluoromount-G Mounting Medium (Invitrogen) containing 10 μg/mL nuclear stain (Hoechst 33342, Invitrogen). Slides were left to air-dry before imaging for FITC using a CELENA X High Content Imaging System. Image processing was done using ImageJ 1.53t.

### Statistical Analysis

Statistical analysis was conducted using GraphPad Prism version 8.0.1. A two-way analysis of variance (ANOVA) test was performed on data on blood glucose levels collected over the study time frame. Data are presented as the mean standard error of the mean (SEM).

## Supporting Information Available

LC-MS chromatogram of FITC insulin and conjugated insulin, protein MS and peptide analysis of the conjugates, NMR characterization of the synthesized compounds (PDF)

## References

[ref1] Purushottam L.; Adusumalli S. R.; Singh U.; Unnikrishnan V. B.; Rawale D. G.; Gujrati M.; Mishra R. K.; Rai V. Single-Site Glycine-Specific Labeling of Proteins. Nat. Commun. 2019, 10 (1), 2539 10.1038/s41467-019-10503-7. 31182711 PMC6557831

[ref2] Ducoux M.; Urbach S.; Baldacci G.; Hübscher U.; Koundrioukoff S.; Christensen J.; Hughes P. Mediation of Proliferating Cell Nuclear Antigen (PCNA)-Dependent DNA Replication through a Conserved P21(Cip1)-like PCNA-Binding Motif Present in the Third Subunit of Human DNA Polymerase Delta. J. Biol. Chem. 2001, 276 (52), 49258–49266. 10.1074/jbc.M106990200. 11595739

[ref3] Gonçalves M. S. T. Fluorescent Labeling of Biomolecules with Organic Probes. Chem. Rev. 2009, 109 (1), 190–212. 10.1021/CR0783840. 19105748

[ref4] Pettinato M. C. Introduction to Antibody-Drug Conjugates. Antibodies 2021, 10 (4), 42 10.3390/antib10040042. 34842621 PMC8628511

[ref5] Lieser R. M.; Yur D.; Sullivan M. O.; Chen W. Site-Specific Bioconjugation Approaches for Enhanced Delivery of Protein Therapeutics and Protein Drug Carriers. Bioconjugate Chem. 2020, 31 (10), 2272–2282. 10.1021/acs.bioconjchem.0c00456. 32931255

[ref6] Bird R. E.; Lemmel S. A.; Yu X.; Zhou Q. A. Bioorthogonal Chemistry and Its Applications. Bioconjugate Chem. 2021, 32 (12), 2457–2479. 10.1021/acs.bioconjchem.1c00461. 34846126

[ref7] Ueno T.; Nagano T. Fluorescent Probes for Sensing and Imaging. Nat. Methods 2011, 8 (8), 642–645. 10.1038/nmeth.1663. 21799499

[ref8] Giepmans B. N. G.; Adams S. R.; Ellisman M. H.; Tsien R. Y. The Fluorescent Toolbox for Assessing Protein Location and Function. Science 2006, 312 (5771), 217–224. 10.1126/science.1124618. 16614209

[ref9] Vira S.; Mekhedov E.; Humphrey G.; Blank P. S. Fluorescent-Labeled Antibodies: Balancing Functionality and Degree of Labeling. Anal. Biochem. 2010, 402 (2), 146–150. 10.1016/j.ab.2010.03.036. 20362543 PMC2876214

[ref10] Cha T. W.; Quo A.; Zhu X. Y. Enzymatic Activity on a Chip: The Critical Role of Protein Orientation. Proteomics 2005, 5 (2), 416–419. 10.1002/PMIC.200400948. 15627963

[ref11] Kalia J.; Raines R. T. Advances in Bioconjugation. Curr. Org. Chem. 2010, 14 (2), 138 10.2174/138527210790069839. 20622973 PMC2901115

[ref12] Ochtrop P.; Hackenberger C. P. R. Recent Advances of Thiol-Selective Bioconjugation Reactions. Curr. Opin. Chem. Biol. 2020, 58, 28–36. 10.1016/j.cbpa.2020.04.017. 32645576

[ref13] Levy D. The Synthesis of Several Tert-Butyloxycarbonyl Derivatives of Insulin. Biochim. Biophys. Acta, Protein Struct. 1973, 328 (1), 107–113. 10.1016/0005-2795(73)90336-X. 4761986

[ref14] Østergaard M.; Mishra N. K.; Jensen K. J. The ABC of Insulin: The Organic Chemistry of a Small Protein. Chem. - Eur. J. 2020, 26 (38), 8341–8357. 10.1002/chem.202000337. 32196765

[ref15] Zhang Y.; Hung-Chieh Chou D. From Natural Insulin to Designed Analogs: A Chemical Biology Exploration. ChemBioChem 2023, 24 (24), e202300470 10.1002/cbic.202300470. 37800626

[ref16] Kolb A. J.; Kaplita P. V.; Hayes D. J.; Park Y. W.; Pernell C.; Major J. S.; Mathis G. Tyrosine Kinase Assays Adapted to Homogeneous Time-Resolved Fluorescence. Drug Discovery Today 1998, 3 (7), 333–342. 10.1016/S1359-6446(98)01204-5.

[ref17] Papathemelis T.; Jablonski E.; Scharl A.; Hauzenberger T.; Gerken M.; Klinkhammer-Schalke M.; Hipp M.; Scharl S. Sentinel Lymph Node Biopsy in Breast Cancer Patients by Means of Indocyanine Green Using the Karl Storz VITOM Fluorescence Camera. BioMed Res. Int. 2018, 2018, 6251468 10.1155/2018/6251468. 29780827 PMC5892256

[ref18] Jameson D. M.; Ross J. A. Fluorescence Polarization/Anisotropy in Diagnostics and Imaging. Chem. Rev. 2010, 110 (5), 2685–2708. 10.1021/cr900267p. 20232898 PMC2868933

[ref19] Banks P. R.; Paquette D. M. Comparison of Three Common Amine Reactive Fluorescent Probes Used for Conjugation to Biomolecules by Capillary Zone Electrophoresis. Bioconjugate Chem. 1995, 6 (4), 447–458. 10.1021/bc00034a015. 7578365

[ref20] Rodríguez-Sáinz C.; Valor L.; Hernández D.; Gil J.; Carbone J.; Pascual-Bernaldez M.; Rodríguez-Alcántara F.; Martínez I.; Vicario J.; Mallal S.; Fernández-Cruz E. Flow Cytometry Analysis with a New FITC-Conjugated Monoclonal Antibody-3E12 for HLA-B*57:01 Rapid Screening in Prevention of Abacavir Hypersensitivity in HIV-1-Infected Patients. HIV Clin. Trials 2013, 14 (4), 160–164. 10.1310/hct1404-160. 23924588

[ref21] Rogers M. V. Light on High-Throughput Screening: Fluorescence-Based Assay Technologies. Drug Discovery Today 1997, 2 (4), 156–160. 10.1016/S1359-6446(97)01016-7.

[ref22] Denora N.; Laquintana V.; Lopalco A.; Iacobazzi R. M.; Lopedota A.; Cutrignelli A.; Iacobellis G.; Annese C.; Cascione M.; Leporatti S.; Franco M. In Vitro Targeting and Imaging the Translocator Protein TSPO 18-KDa through G(4)-PAMAM–FITC Labeled Dendrimer. J. Controlled Release 2013, 172 (3), 1111–1125. 10.1016/j.jconrel.2013.09.024. 24096015

[ref23] Grimm J. B.; Lavis L. D. Caveat Fluorophore: An Insiders’ Guide to Small-Molecule Fluorescent Labels. Nat. Methods 2021, 19 (2), 149–158. 10.1038/s41592-021-01338-6. 34949811

[ref24] Jacob D.; Joan Taylor M.; Tomlins P.; Sahota T. S. Synthesis and Identification of FITC-Insulin Conjugates Produced Using Human Insulin and Insulin Analogues for Biomedical Applications. J. Fluoresc. 2016, 26 (2), 617–629. 10.1007/s10895-015-1748-1. 26658795

[ref25] Ciencialová A.; Žáková L.; Jiráček J.; Barthová J.; Barth T. Preparation and Characterization of Two LysB29 Specifically Labelled Fluorescent Derivatives of Human Insulin. J. Pept. Sci. 2004, 10 (7), 470–478. 10.1002/psc.556. 15298182

[ref26] Niu M.; Tan Y.; Guan P.; Hovgaard L.; Lu Y.; Qi J.; Lian R.; Li X.; Wu W. Enhanced Oral Absorption of Insulin-Loaded Liposomes Containing Bile Salts: A Mechanistic Study. Int. J. Pharm. 2014, 460 (1–2), 119–130. 10.1016/j.ijpharm.2013.11.028. 24275447

[ref27] Elsayed A. M.; Khaled A. H.; Al Remawi M. M.; Qinna N. A.; Farsakh H. A.; Badwan A. A. Low Molecular Weight Chitosan-Insulin Complexes Solubilized in a Mixture of Self-Assembled Labrosol and Plurol Oleaque and Their Glucose Reduction Activity in Rats. Mar. Drugs 2018, 16 (1), 32 10.3390/MD16010032. 29337857 PMC5793080

[ref28] Hentz N. G.; Richardson J. M.; Sportsman J. R.; et al. Synthesis and Characterization of Insulin-Fluorescein Derivatives for Bioanalytical Applications. Anal. Chem. 1997, 69 (24), 4994–5000. 10.1021/ac970726m. 9414613

[ref29] Wang T.; Riegger A.; Lamla M.; Wiese S.; Oeckl P.; Otto M.; Wu Y.; Fischer S.; Barth H.; Ling Kuan S.; Weil T. Water-Soluble Allyl Sulfones for Dual Site-Specific Labelling of Proteins and Cyclic Peptides. Chem. Sci. 2016, 7 (5), 3234–3239. 10.1039/C6SC00005C. 29997815 PMC6006486

[ref30] Hentz N. G.; Richardson J. M.; Sportsman J. R.; Daijo J.; Sittampalam G. S. Synthesis and Characterization of Insulin–Fluorescein Derivatives for Bioanalytical Applications. Anal. Chem. 1997, 69 (24), 4994–5000. 10.1021/AC970726M. 9414613

[ref31] Shah D.; Guo Y.; Ocando J.; Shao J. FITC Labeling of Human Insulin and Transport of FITC-Insulin Conjugates through MDCK Cell Monolayer. J. Pharm. Anal. 2019, 9 (6), 400–405. 10.1016/j.jpha.2019.08.002. 31890339 PMC6931083

[ref32] Bromer W. W.; Sheehan S. K.; Berns A. W.; Arquilla E. R. Preparation and Properties of Fluoresceinthiocarbamyl Insulins. Biochemistry 1967, 6 (8), 2378–2388. 10.1021/bi00860a013. 6049463

[ref33] Pullen R. A.; Lindsay D. G.; Wood S. P.; Tickle I. J.; Blundell T. L.; Wollmer A.; Krail G.; Brandenburg D.; Zahn H.; Gliemann J.; Gammeltoft S. Receptor-Binding Region of Insulin. Nature 1976, 259 (5542), 369–373. 10.1038/259369a0. 175286

[ref34] Bailey I. A.; Garratt C. J.; Penzer G. R.; Smith D. S. The Interaction of B29-Fluoresceinthiocarbamyl-Insulin with Adipocyte Membranes. FEBS Lett. 1980, 121 (2), 246–248. 10.1016/0014-5793(80)80353-X. 7007080

[ref35] Ma Z.; Lim L. Y. Uptake of Chitosan and Associated Insulin in Caco-2 Cell Monolayers: A Comparison between Chitosan Molecules and Chitosan Nanoparticles. Pharm. Res. 2003, 20 (11), 1812–1819. 10.1023/B:PHAM.0000003379.76417.3E/METRICS. 14661926

[ref36] Tsai Y. J.; Rottero A.; Chow D. D.; Hwang K. J.; Lee V. H. L.; Zhu G.; Chan K. K. Synthesis and Purification of NB1-Palmitoyl Insulin. J. Pharm. Sci. 1997, 86 (11), 1264–1268. 10.1021/JS9701263. 9383737

[ref37] Meldal M.; Diness F. Recent Fascinating Aspects of the CuAAC Click Reaction. Trends Chem. 2020, 2 (6), 569–584. 10.1016/j.trechm.2020.03.007.

[ref38] Chen D.; Disotuar M. M.; Xiong X.; Wang Y.; Chou D. H.-C. Selective N-Terminal Functionalization of Native Peptides and Proteins. Chem. Sci. 2017, 8 (4), 2717–2722. 10.1039/C6SC04744K. 28553506 PMC5426342

[ref39] Weissleder R.; Tung C. H.; Mahmood U.; Bogdanov A. In Vivo Imaging of Tumors with Protease-Activated near-Infrared Fluorescent Probes. Nat. Biotechnol. 1999, 17 (4), 375–378. 10.1038/7933. 10207887

[ref40] Chen X.; Conti P. S.; Moats R. A. In Vivo Near-Infrared Fluorescence Imaging of Integrin Avβ3 in Brain Tumor Xenografts. Cancer Res. 2004, 64 (21), 8009–8014. 10.1158/0008-5472.CAN-04-1956. 15520209

[ref41] Myochin T.; Hanaoka K.; Komatsu T.; Terai T.; Nagano T. Design Strategy for a Near-Infrared Fluorescence Probe for Matrix Metalloproteinase Utilizing Highly Cell Permeable Boron Dipyrromethene. J. Am. Chem. Soc. 2012, 134 (33), 13730–13737. 10.1021/ja303931b. 22830429

[ref42] Miao Z.; Ren G.; Liu H.; Jiang L.; Cheng Z. Cy5.5-Labeled Affibody Molecule for near-Infrared Fluorescent Optical Imaging of Epidermal Growth Factor Receptor Positive Tumors. J. Biomed. Opt. 2010, 15 (3), 036007 10.1117/1.3432738. 20615009

[ref43] Ballou B.; Fisher G. W.; Deng J. S.; Hakala T. R.; Srivastava M.; Farkas D. L. Cyanine Fluorochrome-Labeled Antibodies in Vivo: Assessment of Tumor Imaging Using Cy3, Cy5, Cy5.5, and Cy7. Cancer Detect. Prev. 1998, 22 (3), 251–257. 10.1046/j.1525-1500.1998.0OA25.x. 9618048

[ref44] Katayama D. S.; Nayar R.; Chou D. K.; Campos J.; Cooper J.; Vander Velde D. G.; Villarete L.; Liu C. P.; Manning M. C. Solution Behavior of a Novel Type 1 Interferon, Interferon-τ. J. Pharm. Sci. 2005, 94 (12), 2703–2715. 10.1002/JPS.20461. 16258985

[ref45] Alkhawaja B.; Al-Akayleh F.; Al-Rubaye Z.; AlDabet G.; Bustami M.; Smairat M.; Agha A. S. A. A.; Nasereddin J.; Qinna N.; Michael A.; Watts A. G. Dissecting the Stability of Atezolizumab with Renewable Amino Acid-Based Ionic Liquids: Colloidal Stability and Anticancer Activity under Thermal Stress. Int. J. Biol. Macromol. 2024, 270, 132208 10.1016/j.ijbiomac.2024.132208. 38723835

[ref46] Chou D. H. C.; Webber M. J.; Tang B. C.; Lin A. B.; Thapa L. S.; Deng D.; Truong J. V.; Cortinas A. B.; Langer R.; Anderson D. G. Glucose-Responsive Insulin Activity by Covalent Modification with Aliphatic Phenylboronic Acid Conjugates. Proc. Natl. Acad. Sci. U.S.A. 2015, 112 (8), 2401–2406. 10.1073/pnas.1424684112. 25675515 PMC4345600

[ref47] Qinna N. A.; Badwan A. A. Impact of Streptozotocin on Altering Normal Glucose Homeostasis during Insulin Testing in Diabetic Rats Compared to Normoglycemic Rats. Drug Des., Dev. Ther. 2015, 9, 2515–2525. 10.2147/DDDT.S79885. PMC4427609 26005328

[ref48] Krentz A. J.; Heinemann L.; Hompesch M. Methods for Quantifying Insulin Sensitivity and Determining Insulin Time-Action Profiles. In Translational Research Methods for Diabetes, Obesity and Cardiometabolic Drug Development; Springer, 2015; pp 3–43.

[ref49] Tuesca A. D.; Reiff C.; Joseph J. I.; Lowman A. M. Synthesis, Characterization and in Vivo Efficacy of PEGylated Insulin for Oral Delivery with Complexation Hydrogels. Pharm. Res. 2009, 26 (3), 727–739. 10.1007/s11095-008-9816-8. 19145407

[ref50] Ma E. L.; Ma H.; Liu Z.; Zheng C. X.; Duan M. X. In Vitro and in Vivo Evaluation of a Novel Oral Insulin Formulation. Acta Pharmacol. Sin. 2006, 27 (10), 1382–1388. 10.1111/j.1745-7254.2006.00424.x. 17007747

[ref51] Sonaje K.; Lin Y. H.; Juang J. H.; Wey S. P.; Chen C. T.; Sung H. W. In Vivo Evaluation of Safety and Efficacy of Self-Assembled Nanoparticles for Oral Insulin Delivery. Biomaterials 2009, 30 (12), 2329–2339. 10.1016/j.biomaterials.2008.12.066. 19176244

