# Supplementary material for: Site-Selective Approaches to Attain Fluorescent Human Insulin Conjugates: Balancing the Site of Labeling and the In Vivo Activity
Source: ACS Omega. 2025 Feb 17;10(8):8140–51. doi: 10.1021/acsomega.4c09498 (PMC11886657; doi:10.1021/acsomega.4c09498)
Supplement: Supplementary file 1 — ao4c09498_si_001.pdf [file ao4c09498_si_001.pdf]

Supporting information:

Site-selective approaches to attain fluorescent human insulin conjugates: Balancing the site of labelling and *the in vivo* activity

Bayan Alkhawaja[1,2*], Ghayda' AlDabet[3], Nour Alkhawaja[1], Bayan Y. Ghanim[3], Khaled Al-Khatib[3], Shaun Reeksting[4], Andreas Michael[2], Duaa Abuarqoub [1,5], Marwa Mohammad[1], Andrew G. Watts[2], Nidal A. Qinna[3*]

[1] Faculty of Pharmacy and Medical Sciences, University of Petra, Amman 11196, Jordan
[2] Department of Life Sciences, University of Bath, Claverton Down, BA2 7AY, Bath, UK
[3] University of Petra Pharmaceutical Center, Faculty of Pharmacy and Medical Sciences, Petra University, Amman 11196, Jordan
[4] Agilent Technologies U.K. Ltd., Lakeside, Cheadle Royal Business Park, Stockport, Cheshire SK8 3GR, UK
[5] Cell Therapy Center, University of Jordan, Amman 11942, Jordan

*Corresponding authors' email:
bayan.alkhawaja@uop.edu.jo
nqinna@uop.edu.jo

Supplementary figures and table

Various equivalents of FITC (1) were used to assess the main fluorescent product and the degree of labelling. Following this, protein mass spectroscopy results were interpreted. Total ion chromatogram (TIC) results demonstrated that performing the reaction over two days at room temperature (RT) with 3 equivalents of fluorescent dye, both di- and tri-labelled insulin adducts were obtained as major products (Figure S1A and B). On the other hand, unlabelled insulin and mono-labelled insulin adducts with no di-FITC or tri-adducts were detected when attempting the reaction with 1.3 equivalents of FITC at 4 ^{o}C (Figure S2).

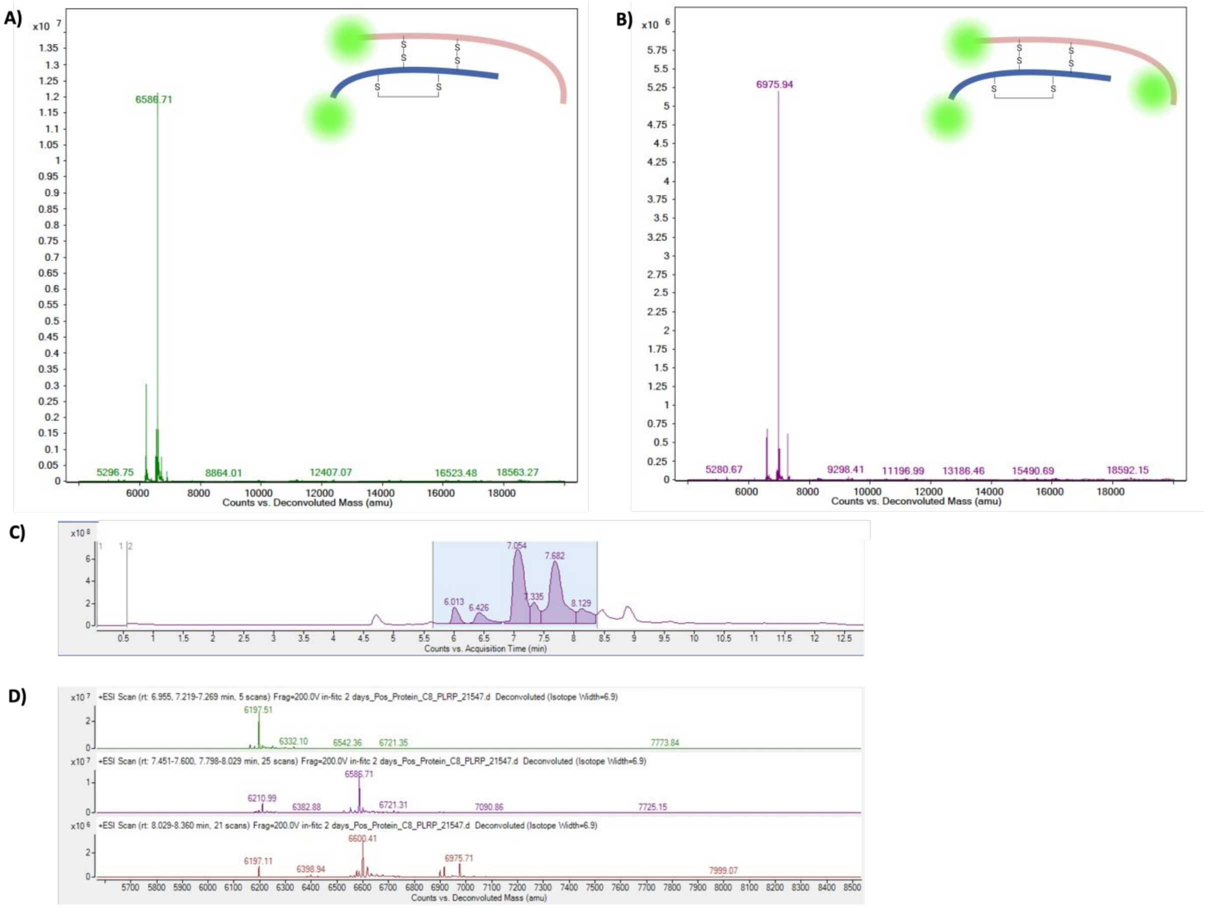

Figure S1. LC-MS chromatogram of FITC insulin. Labelling insulin with excess FITC affording A) (Gly^{A1}, Phe^{B1}-N) di-FITC, B) (Gly^{A1}, Phe^{B1}Lys^{B29}-N) tri-FITC insulin adducts, C) chromatogram results and D) deconvolution results.

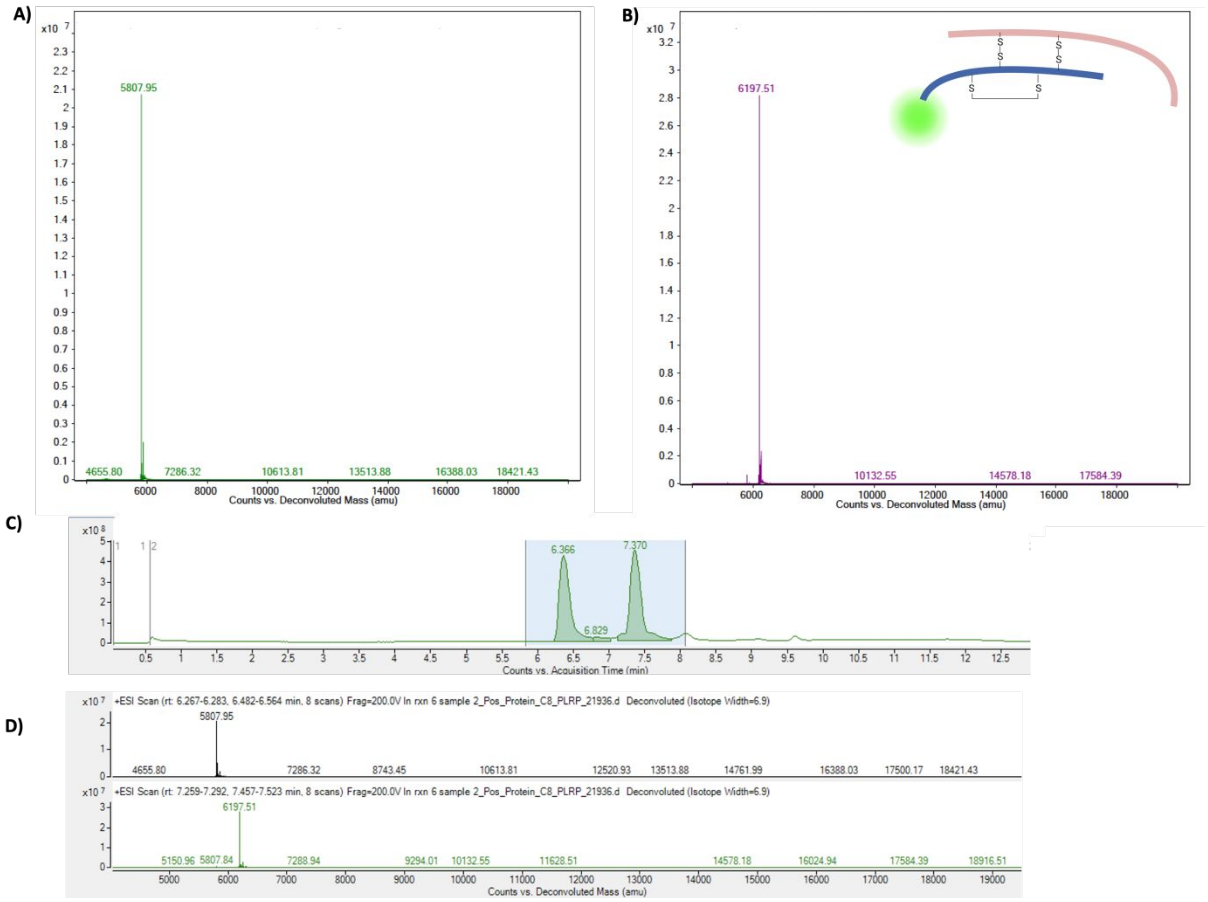

Figure S2. LC-MS chromatogram of FITC insulin. Labelling insulin with limited equiv. of FITC at 4 °C, giving A) unmodified Insulin, B) Gly^{A1}-N-FITC insulin adducts C) chromatogram results and D) deconvolution results.

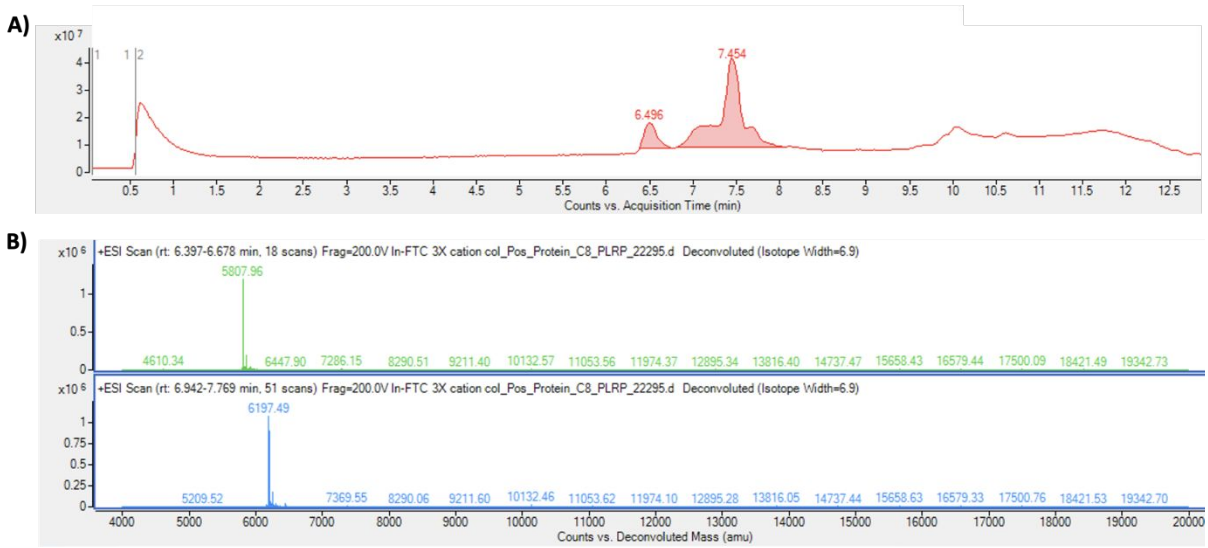

Figure S3. LC-MS chromatogram of FITC-insulin. Labelling insulin with limited equiv. of FITC at RT, affording mainly Gly^{A1}-N-FITC insulin adduct. A) chromatogram results and B) deconvolution results.

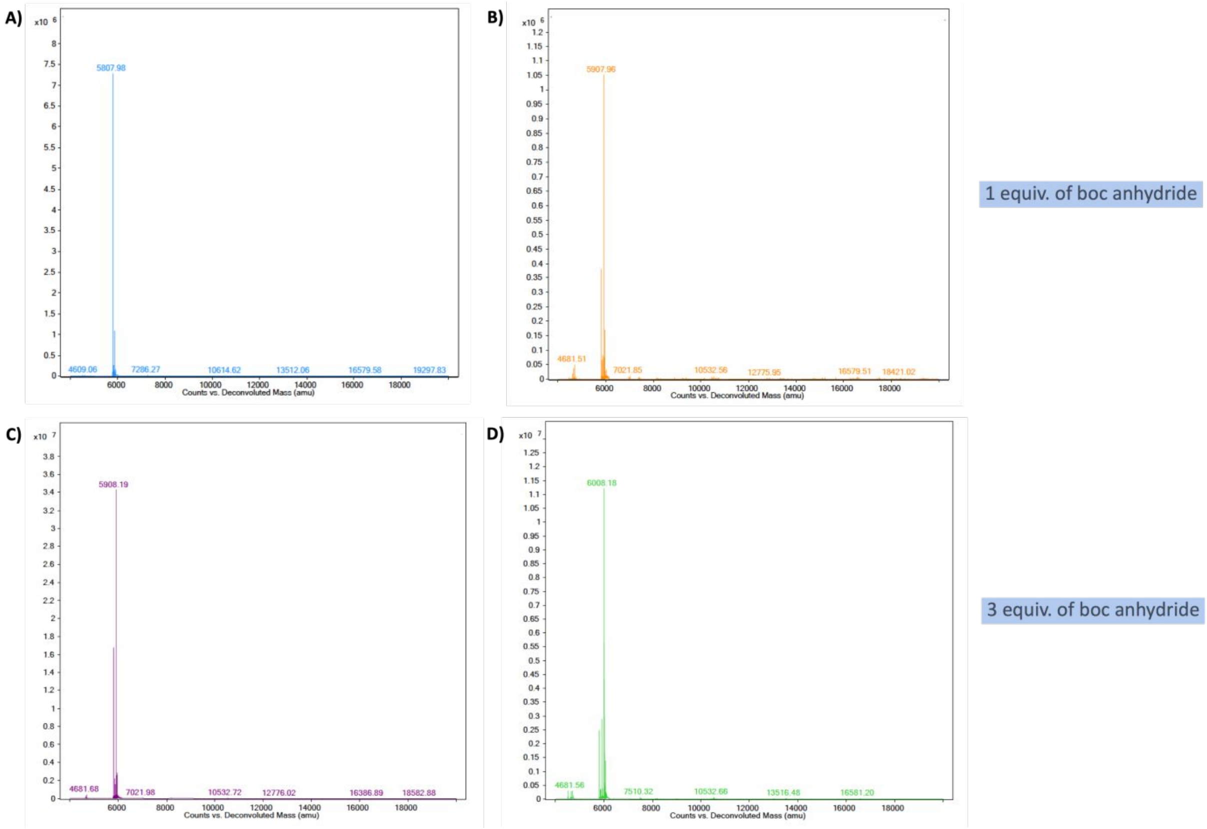

Figure S4. LC-MS chromatogram of conjugated insulin. Attained products of labelling insulin with various equiv. of boc anhydride. **A&B**) 1 equiv. of boc anhydride. **C&D**) 3 equiv. of Boc anhydride.

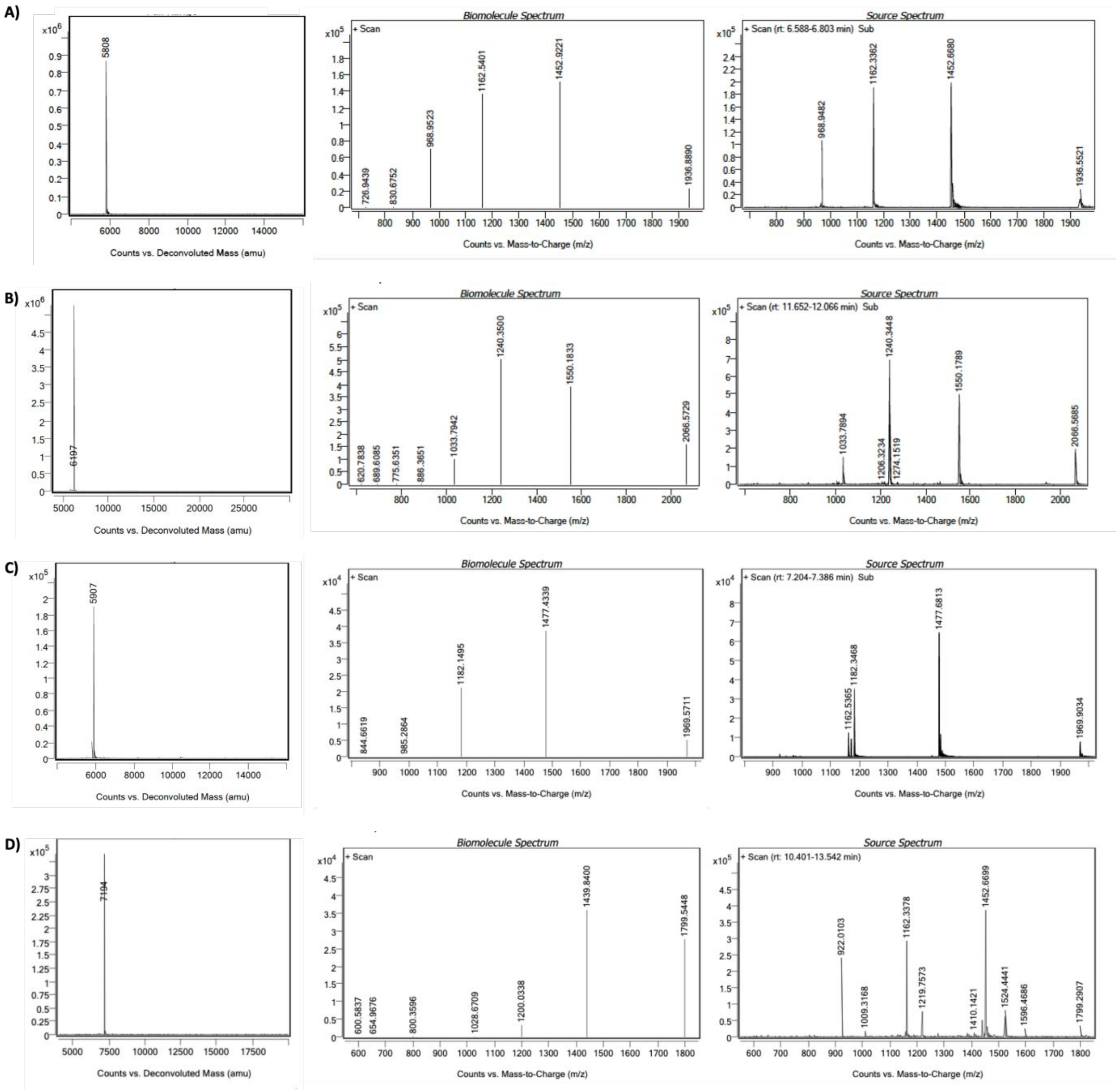

Figure S5. Deconvoluted protein spectra (left), showing the clean (middle) and raw-multiple charge state (right) species, of A) Insulin, B) Gly^{A1}-N-FITC-Insulin, C) Gly^{A1}-N-boc-Insulin, D) Gly^{A1}-N-Cy5-Insulin

Table S1. Protein MS and peptide analysis of the conjugates

Conjugates	Calculated molecular weight of labelled insulin (Da)	Found MS (Da)	Calculated molecular weight of Peptide chain (Da)	Peptide peak (*m/z*)
Insulin Gly^{A1}-N-FITC	6197.69	6197.62	Chain A+FITC: 2773.28	$(1409.5+2Na)^{2+}$
Insulin Gly^{A1}-N-Boc	5909.39	5908.10	Chain A+ Boc: 2483.8	$(1264.58+2Na)^{2+}$
Insulin Gly^{A1}-NBoc, Phe ^{B1}-N-FITC	6297.39	6297.51	Chain B+FITC: 3819.53	$(955.69+4H)^{4+}$
Insulin Gly^{A1}-NN_3	6185.58	6185.49		-
Insulin Gly^{A1}-NCy5	7193.18	7194.54		-

NMR Characterisation

*4-(2-chloroacetamido)benzoic acid (**a**)*

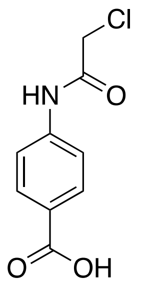

(a)

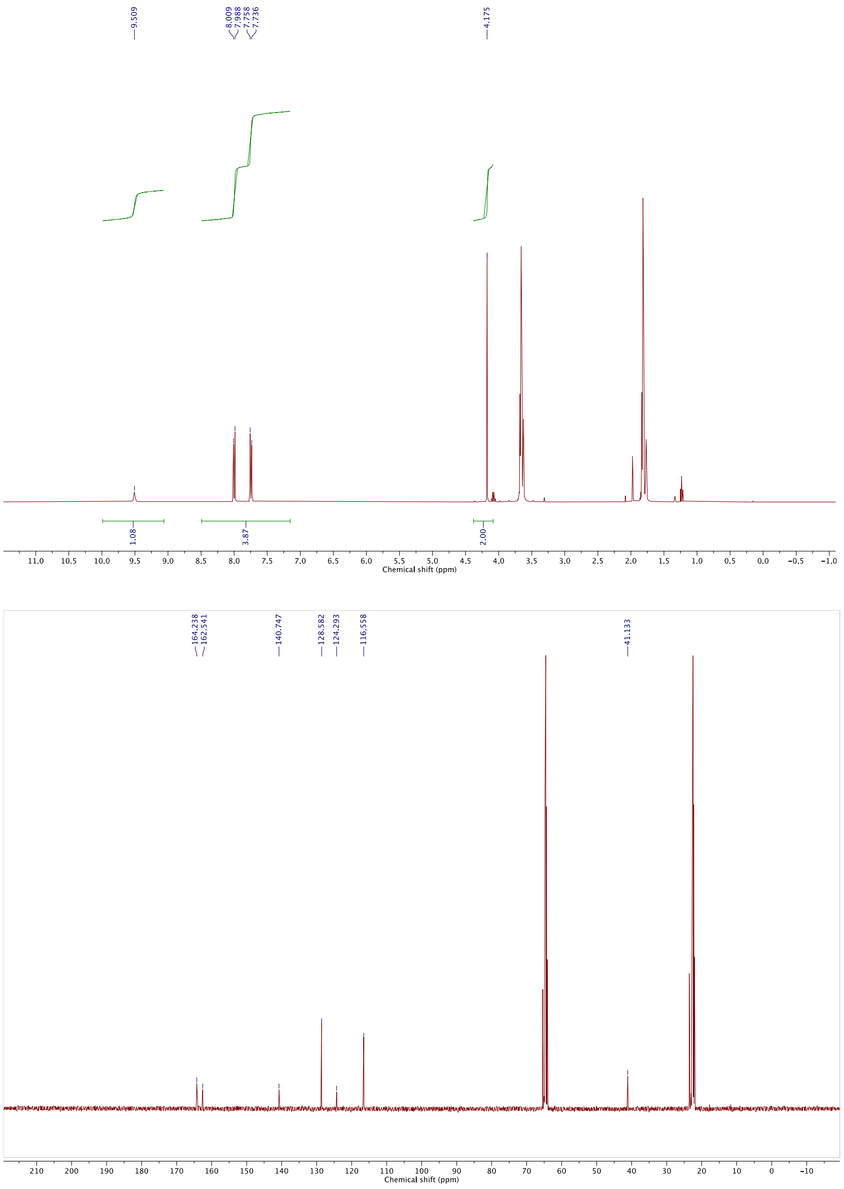

Figure S6. ^{1}H and ^{13}C NMR spectra of *4-(2-chloroacetamido)benzoic acid (**a**)*

N-(2-(2-(2-(2-azidoethoxy)ethoxy)ethoxy)ethyl)-4-(2-chloroacetamido)benzamide (**c**)

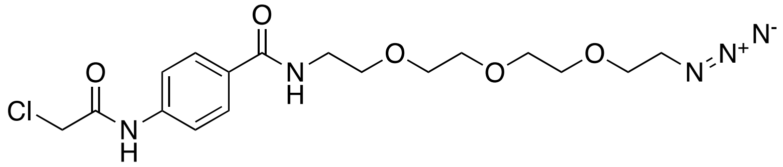

(c)

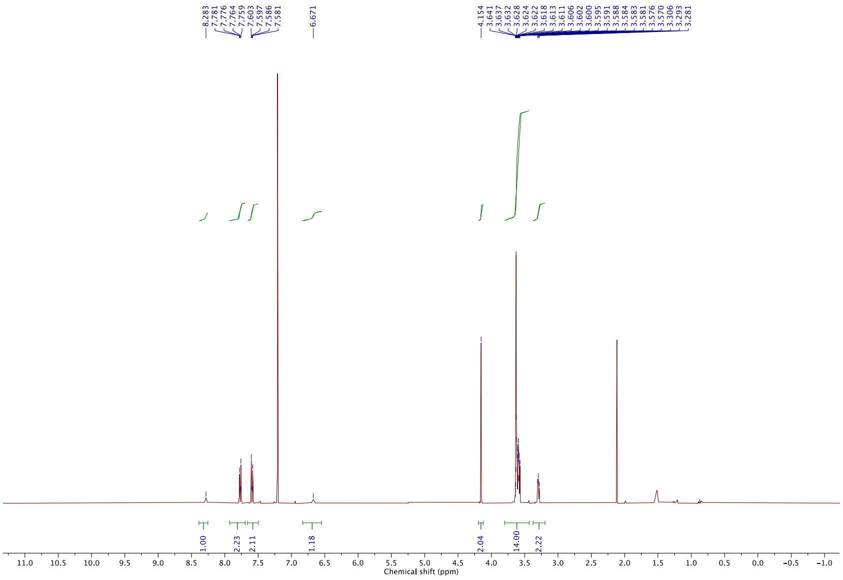

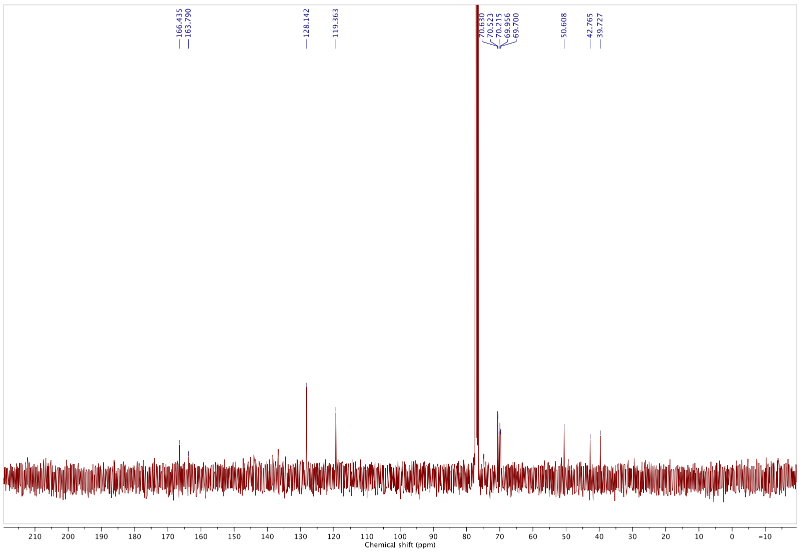

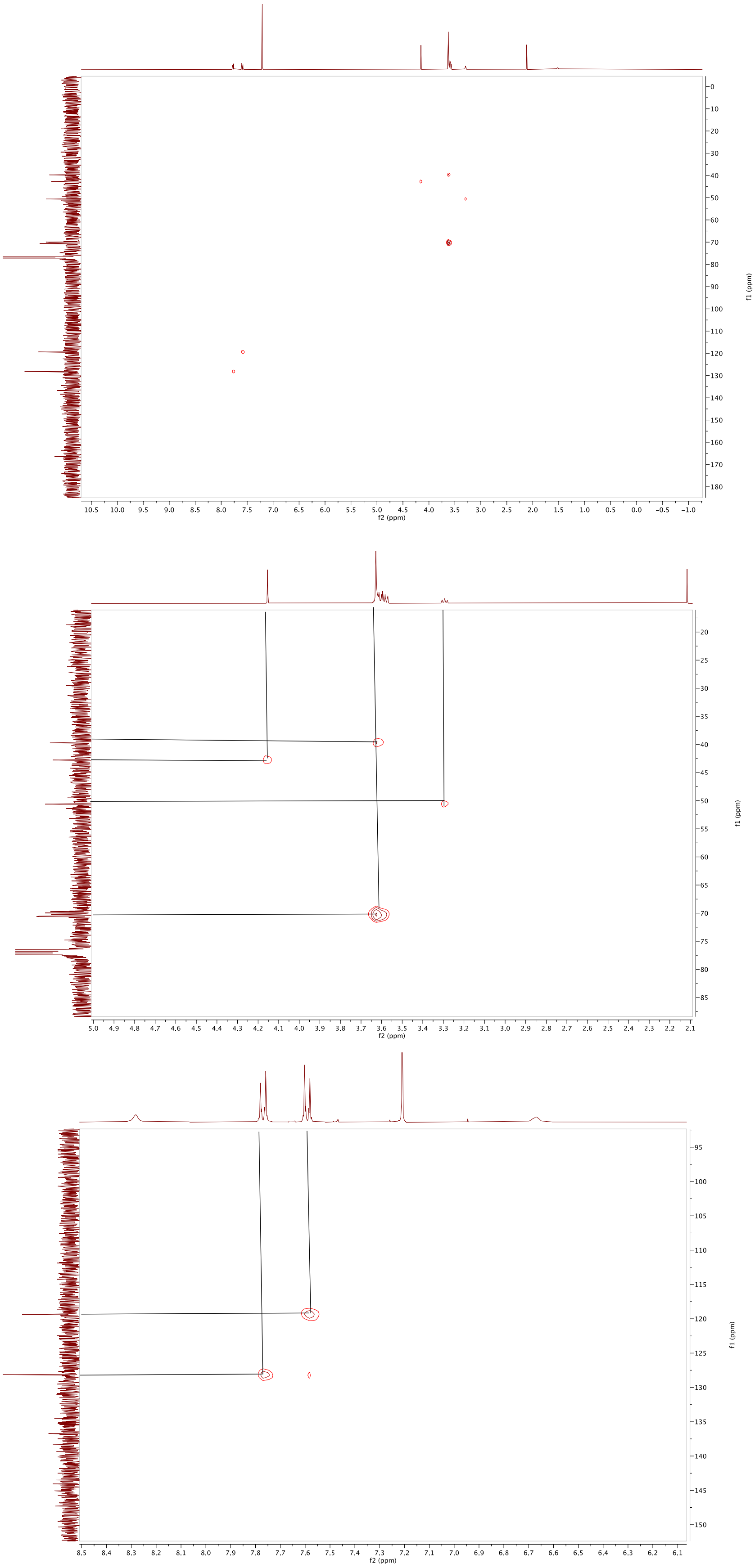

Figure S7. ^{1}H, ^{13}C and HSQC NMR spectra of N-(2-(2-(2-(2-azidoethoxy)ethoxy)ethoxy)ethyl)-4-(2-chloroacetamido)benzamide (**c**)

N-(2-(2-(2-(2-azidoethoxy)ethoxy)ethoxy)ethyl)-4-(2-iodoacetamido)benzamide ***(3)***

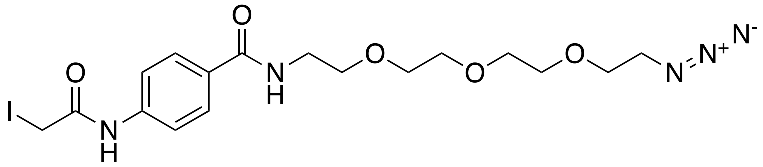

(3)

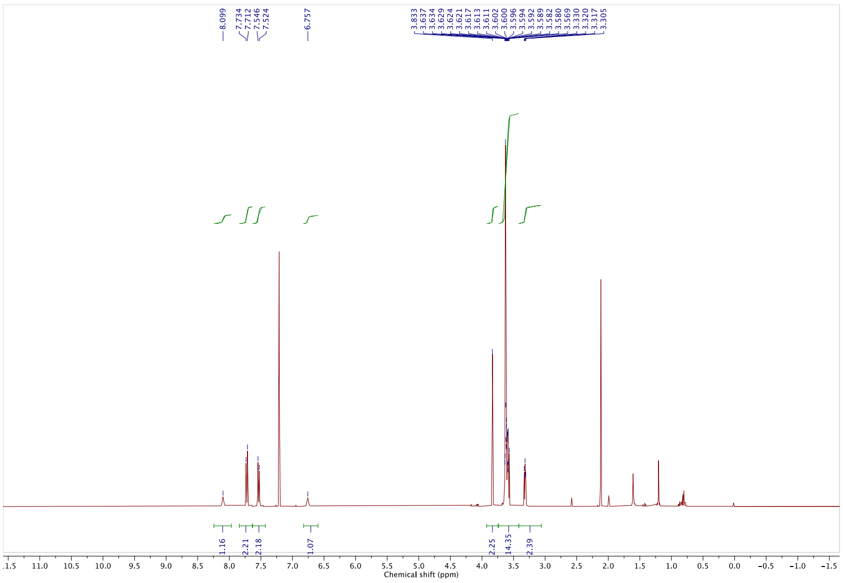

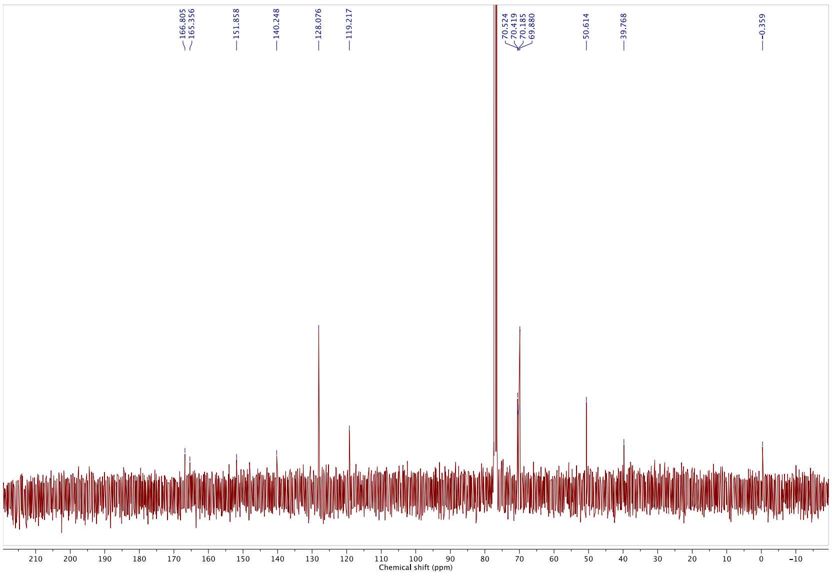

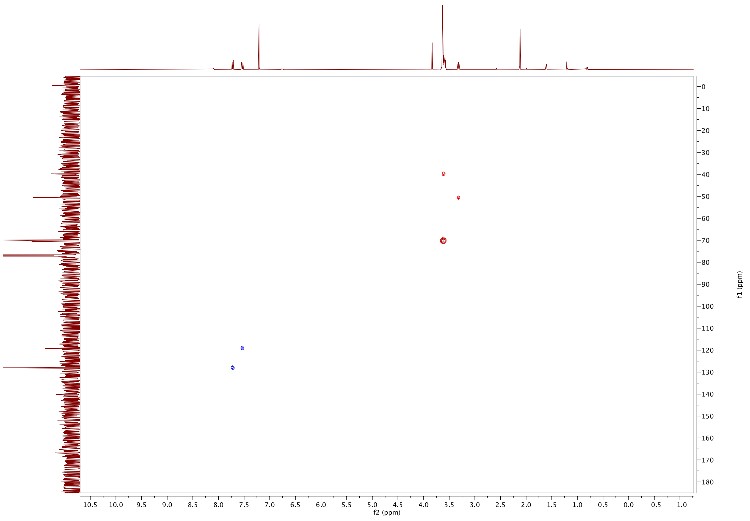

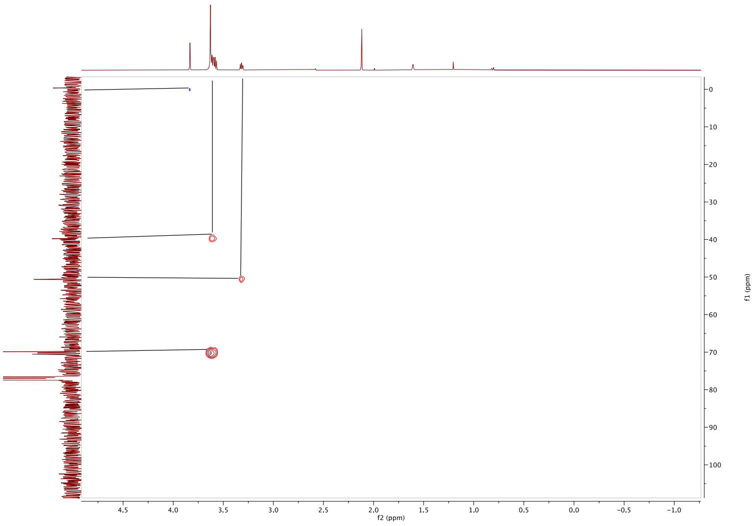

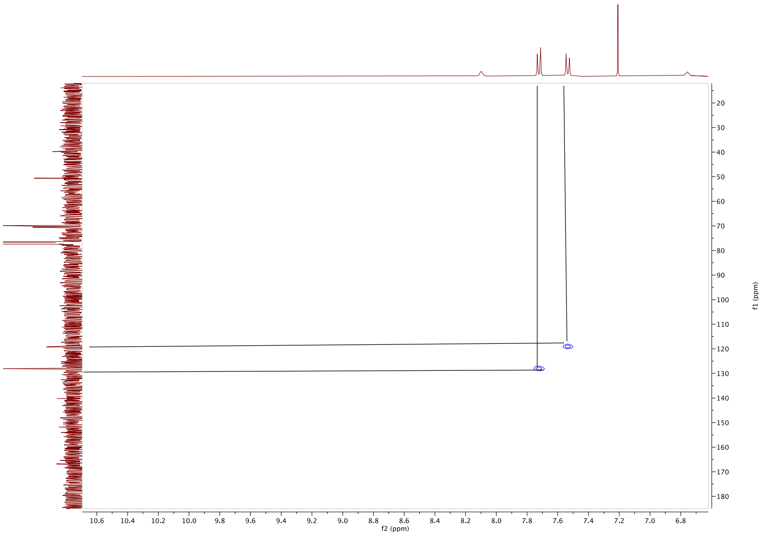

Figure S8. ^{1}H, ^{13}C and HSQC NMR spectra of N-(2-(2-(2-(2-azidoethoxy)ethoxy)ethoxy)ethyl)-4-(2-iodoacetamido)benzamide (**3**)
